# Unveiling Hidden Intramolecular Non‐Covalent Interactions in a Neutral Serine, Its Zwitterion, Cluster, and Crystal by Features of Electron Density

**DOI:** 10.1002/jcc.70134

**Published:** 2025-06-30

**Authors:** Vasilii Korotenko, Anna Egorova, Vladimir Tsirelson

**Affiliations:** ^1^ Thermal Separation Processes Hamburg University of Technology (TUHH) Hamburg Germany; ^2^ Department of Chemistry LMU München München Germany; ^3^ Quantum Chemistry Department D. I. Mendeleev University Moscow Russia

**Keywords:** crystal, electron density, electron pressure, IQA, molecular cluster, non‐covalent interactions, QTAIM, RDG, serine, zwitterion

## Abstract

We investigate intramolecular non‐covalent interactions (NCIs) in neutral serine, its zwitterion, molecular clusters, and crystal using electron density‐based approaches, including QTAIM, RDG, IQA, and electronic pressure analysis. In addition to completed NCIs (hydrogen bonds with bond paths), we identify latent interactions—attractive, bond‐path‐free atomic pair interactions with negative interaction energies. These are classified into *dynamic* (vibration‐induced and transient) and *static* (secondary, persistent but structurally passive) types. Analysis of the internal pressure in electronic continuum reveals that latent NCIs exhibit distinct signatures in the kinetic and exchange components, which evolve across the molecular, cluster, and crystalline states. *Dynamic* interactions are characterized by off‐axis minima in the exchange part of the pressure, whereas *static* interactions lack such features. Upon crystallization, intramolecular latent NCIs may disappear due to electron density redistribution and the formation of intermolecular hydrogen bonds. These intermolecular contacts may also spatially constrain atoms, suppressing vibrational flexibility and effectively converting *dynamic* NCIs into *static* ones. The kinetic pressure highlights regions of electron localization, while the exchange pressure offers a physical criterion for distinguishing different types of NCIs. Our findings demonstrate the structural and stabilizing roles of latent interactions and establish electronic pressure as a sensitive and informative descriptor for their analysis.

## Graphical abstract

We reveal a new class of intramolecular noncovalent interactions called “latent” that lack bond paths yet stabilize molecules through subtle electronic effects. Using electron density and electronic pressure analysis, we classify them as “dynamic” or “static” and track their evolution across isolated molecules, molecular clusters, and crystals.

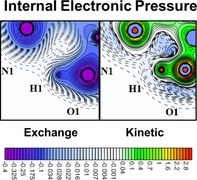

**Abbreviations**
ED: electron density
IQA: interacting quantum atoms
NCI: non‐covalent interaction
QTAIMC: quantum theory of atoms in molecules and crystals
RDG: reduced density gradient

## 1 Introduction

Non‐covalent interactions (NCIs), those quiet but powerful forces, play a crucial role in biomolecular systems [1, 2], contributing to protein folding processes [3, 4, 5, 6], substrate–enzyme “lock‐and‐key” recognition [7, 8], and drug action mechanisms. Structural models of living system components—molecules, clusters of simple amino acids, and molecular crystals—are essential for understanding these phenomena. Unfortunately, experimental studies of such systems are often limited by their conformational flexibility and low thermal stability. As a result, reliable structural data exist primarily for the simplest aliphatic amino acids [9, 10, 11, 12, 13, 14, 15, 16, 17, 18, 19, 20, 21]. This is where quantum chemistry steps in—not as a luxury, but as a necessity.

**FIGURE 1:**
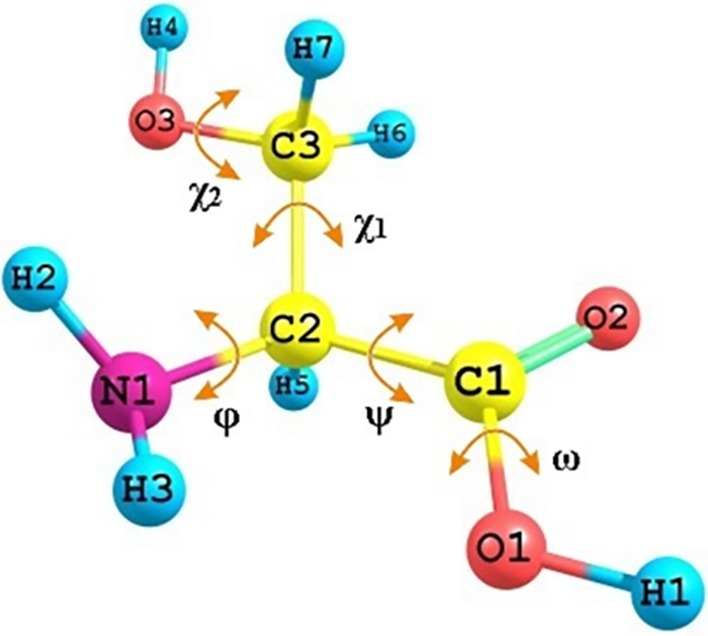
Atom numbering and dihedral angles of the neutral serine molecule: *φ* = (H2‐N1‐C2‐C1); *ψ* = (N1‐C2‐C1‐O1); *ω* = (C2‐C1‐O1‐H1); *χ*_1_ = (N1‐C2‐C3‐O3); *χ*_2_ = (C2‐C3‐O3‐H4).

Serine (NH_2_–CHCH_2_OH–COOH), a nonessential amino acid (2‐amino‐3‐hydroxypropanoic acid), is a constituent of nearly all natural proteins and contributes to the formation of active sites in esterases and peptidases [22]. Like most amino acids, serine exists in a nonionized form in the gas phase [18, 21, 23, 24, 25, 26], featuring four proton donor and four proton acceptor groups (Figure 1). In condensed phases (solution or crystal), the molecule adopts a zwitterionic form [26, 27]. These structural features, combined with its biological relevance and chemical simplicity, make serine a particularly suitable subject for the investigation of both intra‐ and intermolecular NCIs using high‐level quantum‐chemical approaches.

NCIs are traditionally identified through interatomic distances, van der Waals radii, and characteristic directionality (linearity) of contacts [27, 28, 29]. However, van der Waals radii are not universal [30], and while strong hydrogen bonds, such as O–H···O, often display clear linearity, weaker interactions like C–H···O tend to deviate from this pattern [31, 32]. This necessitates the use of complementary approaches that go beyond purely geometric criteria and involve the analysis of electron density (ED).

Moreover, many observed binding features in polyatomic molecules, clusters, and crystals do not fully align with existing theoretical frameworks of chemical bonding [33]. This issue will be discussed in more detail below. Developing a consistent and unified description of various types of atomic and molecular interactions remains an open and important challenge in structural chemistry.

The aim of this work is to investigate intra‐ and intermolecular NCIs in neutral serine conformers, its zwitterionic form, a molecular cluster, and the serine crystal, based on the analysis of ED obtained from both theoretical calculations and X‐ray diffraction data. To achieve this, we employ a combination of complementary methods for identifying and characterizing NCIs, all relying on the properties of the ED distribution.

## 2 Methodology

The ED, *ρ*(*r*), is a scalar function related to the many‐electron wave function, Ψ, by the relationship:

(1) $$\begin{array}{c} \rho \left(r_{0}\right)=\left\langle \Psi |e\sum_{i}^{N}\delta \left(r-r_{i}\right)|\Psi \right\rangle \\ \;=e\ldots \int_{s_{1}}\Psi^{*}\left(r_{1},s_{1},\ldots ,r_{N},s_{N}\right)\\ \;\times \sum_{i}^{N}\delta \left(r-r_{i}\right)\Psi \left(r_{1},s_{1},\ldots ,r_{N},s_{N}\right){\mathrm{dr}}_{1}{\mathrm{ds}}_{1}\ldots \\ \;=\mathrm{Ne}\int_{r_{2}}\ldots \int_{s_{1}}\Psi^{*}\left(r_{1},s_{1},\ldots ,r_{N},s_{N}\right)\\ \;\times \Psi \left(r_{1},s_{1},\ldots ,r_{N},s_{N}\right)dr_{2}{\mathrm{ds}}_{1}\ldots \end{array}$$

where $e\sum_{i}^{N}\delta \left(r-r_{i}\right)$ the operator of the local ED at point $r_{i}$, *e* is the electron charge. The variables *s*_𝑖_ denote the spin coordinates of electrons. Integration over the spin variables in Equation (1) is equivalent to summation over spin orientations. Due to the indistinguishability of electrons, summation over electrons in Equation (1) is replaced by multiplying by the total number of electrons *N*. From Equation (1), it follows that the ED represents the average density of *N* electrons in their ground state, averaged over inter‐electronic interactions. The ED depends only on the spatial coordinate **r**.

A direct approach to analyzing ED is provided by the quantum topological theory of molecular and crystalline structure, developed by Bader and collaborators [34]. This theory, known as the Quantum Topology of Atoms in Molecules and Crystals (QTAIMC), is based on the topological analysis of the ED distribution function *ρ*(**r**). The origin of this function can vary: it may be derived experimentally from coherent X‐ray diffraction or through quantum‐chemical calculations.

The gradient of the ED equals zero at critical points (CP):

(2) $$\nabla \rho \left(r\right)=0\;\text{or}\;\frac{\partial \rho }{\mathrm{\partial x}}=\frac{\partial \rho }{\mathrm{\partial y}}=\frac{\partial \rho }{\mathrm{\partial z}}=0$$

The second derivatives of the ED, calculated at these points, form a real symmetric 3 × 3 Hessian matrix. Its eigenvalues $\lambda_{x}$, $\lambda_{y}$, $\lambda_{z}$, are then sorted in ascending order, and their values are renumbered accordingly: the principal curvatures of the ED at the CP are denoted as $\lambda_{1}\leq \lambda_{2}\leq \lambda_{3}$. These eigenvalues determine the rank *p* and the signature *q* of the CP (*p*, *q*). The rank *p* = 3 corresponds to the number of non‐zero eigenvalues $\lambda_{i}$, while *q* is the algebraic sum of the signs of the eigenvalues $\lambda_{i}$ at the CP. In the ED, four nondegenerate CPs are possible: (3, −3), known as an attractor; (3, −1), representing a BCP; (3, +1), describing a ring CP; and (3, +3), corresponding to a cage CP [34, 35]. Each of these CPs corresponds to specific structural elements of a molecule or crystal: atomic positions, bonds between atoms, ring and cage fragments of a structure.

In QTAIMC, the boundaries of atoms in molecules are defined as closed surfaces enclosing each nucleus. The flux of the gradient vector of the ED through these surfaces is equal to zero at every point:

(3) $$\nabla \rho \left(r\right)\cdot n\left(r\right)=0;\forall r\in S\left(r\right)$$

$n\left(r\right)$ is the unit vector normal to the surface at the point $S\left(r\right)$. Thanks to condition in Equation (3), any interatomic surface is formed by gradient trajectories that end at a bond CP located on this surface.

Interactions between atoms in molecules and crystals are associated with bond paths in the ED: bridges of ED, represented by pairs of gradient lines that start at the same bond CP and end at nuclei. The presence of bond paths between a pair of atoms (or a bond CP) in the interatomic space is postulated in QTAIMC as a necessary and sufficient condition for the existence of a bonding chemical interaction between these atoms in the equilibrium state [35, 36, 37]. Such interactions are naturally regarded as *structure‐forming or completed*.

Low values of the ED and its reduced density gradient (RDG)

(4) $$\mathrm{RDG}\left(r\right)=\frac{1}{2{\left(3\pi^{2}\right)}^{\frac{1}{3}}}*\frac{|\nabla \rho \left(r\right)|}{\rho {\left(r\right)}^{\frac{4}{3}}}$$

identify regions between pairs of atoms where a bond path may occur (a point where RDG = 0 exists) [38, 39, 40]. Isosurfaces of RDG encompassing a molecule or the area between two interacting atoms are always closed. In the latter case, this is caused by the accumulation of ED in the interatomic space and the local slowing of the exponential decay of the ED as the distance from the molecule increases. As proposed [38], the function sign[*λ*_2_(*r*)]*ρ(*r*) is also analyzed, where *λ*_2_ *(r)* is the second‐largest eigenvalue of the Hessian matrix of the ED. The sign of *λ*_2_(*r*) helps identify different types of NCIs [35, 37]. The combination of descriptors RDG(r) and sign[*λ*_2_(*r*)]*ρ(*r*), characterizes the curvature of the ED at point r along one of the orthogonal directions in the interatomic space. Regions with low values of ED and its RDG are associated with NCIs. However, the presence of a bond path in these regions is not mandatory. In this case, one can refer to *potentially possible* atomic interactions, which are proposed to be called *incomplete* [41] or *latent* (hidden) interactions.

In some cases, even in the absence of bond paths, the distribution of ED may be such that small deviations of nuclei from equilibrium due to vibrations can lead to the appearance of “pseudo‐bond paths”—bridges of ED analogous to bond paths in equilibrium systems. Thus, during conformational changes or under favorable conditions of atomic vibrations in real systems, a “pulsating” network of atomic contacts may exist, in which bond paths or pseudo‐bond paths can form or break. Such atomic “contacts” will be referred to as *dynamic* latent interactions. NCIs between atoms for which pseudo‐bond paths do not form in structures resulting from atomic vibrations will be classified as *static latent* (secondary) interactions. We employ vibrational frequency analysis and the corresponding normal mode analysis to identify NCIs in the serine molecule. This approach is based on the calculation of the Hessian matrix of ED and its diagonalization, which allows us to determine the normal modes of vibration using the rigid rotor/harmonic oscillator model [42]. Each normal mode represents a collective atomic motion describing their mutual displacements within the harmonic approximation [43]. Of particular interest to us are modes in which coordinated atomic motions indicate hidden NCIs [44, 45, 46]. For example, hydrogen bonds manifest as low‐frequency stretching vibrations of hydrogen and acceptor atoms, while weak long‐range interactions can induce correlated atomic displacements even in the absence of an explicit bonding pathway. The analysis of these modes not only confirms the presence of expected hydrogen bonds but also reveals additional *latent* NCIs that are difficult to identify based on geometric criteria alone.

The nature of NCIs can be further detailed using the Interacting Quantum Atoms (IQA) method [47, 48, 49, 50, 51]. The IQA method is based on identifying pairwise energy contributions to atomic interactions by partitioning the complex system—a molecule or a complex—into atomic basins, which, according to QTAIMC, are separated by zero‐flux surfaces. The conceptual distinction between IQA and QTAIMC lies in the fact that IQA does not rely on the concept of bond paths. Instead, it is based on the principle that all pairs of atoms in a system interact due to the delocalization of electronic wave functions. The energetic characteristics of these interactions are obtained by integrating corresponding energy operators over pairs of atomic basins.

For each pair of atoms A and B, the contributions to the total energy may be divided into intra‐atomic energy $E_{\text{intra}}^{i}$, where *i* = A, B, and the interatomic interaction energy $E_{\text{inter}}^{\mathrm{AB}}$:

$$E_{\text{intra}}^{A}=T^{A}+V_{\mathrm{en}}^{\mathrm{AA}}+V_{\mathrm{ee}}^{\mathrm{AA}}$$

$$E_{\text{inter}}^{\mathrm{AB}}=V_{\mathrm{nn}}^{\mathrm{AB}}+V_{\mathrm{en}}^{\mathrm{AB}}+V_{\mathrm{ne}}^{\mathrm{AB}}+V_{\mathrm{ee}}^{\mathrm{AB}},А\neq B$$

Here, $T^{A}$ represents the kinetic energy, $V_{\mathrm{nn}}^{\mathrm{AB}}$ the nucleus‐nucleus interaction energy, $V_{\mathrm{en}}^{\mathrm{AB}}$ and $V_{\mathrm{ne}}^{\mathrm{AB}}$ the electron‐nucleus interaction energy, and $V_{\mathrm{ee}}^{\mathrm{AB}}$ the electron–electron interaction energy. The contribution $V_{\mathrm{ee}}^{\mathrm{AB}}$ is further divided into electrostatic ($V_{\mathrm{els}}^{\mathrm{AB}}$), exchange ($V_{X}^{\mathrm{AB}}$), and correlation components ($V_{C}^{\mathrm{AB}}$):

$$V_{\mathrm{ee}}^{\mathrm{AB}}=V_{\mathrm{els}}^{\mathrm{AB}}+V_{X}^{\mathrm{AB}}+V_{C}^{\mathrm{AB}},$$

The total electrostatic interaction energy $V_{\mathrm{els}}^{\mathrm{AB}}$ is given by

$$V_{\mathrm{els}}^{\mathrm{AB}}=V_{\mathrm{nn}}^{\mathrm{AB}}+V_{\mathrm{ne}}^{\mathrm{AB}}+V_{\mathrm{en}}^{\mathrm{AB}}+V_{\text{Coul}}^{\mathrm{AB}}$$

The total energy of interatomic interaction is defined as the sum of one‐atom and two‐atom contributions:

$$E_{\mathrm{int}}=\sum_{A}E_{\text{intra}}^{A}+\frac{1}{2}\sum_{A}\sum_{B\neq А}E_{\text{inter}}^{\mathrm{AB}}$$

where

$$E_{\text{inter}}^{\mathrm{AB}}=E_{\mathrm{els}}^{\mathrm{AB}}+E_{X}^{\mathrm{AB}}+E_{C}^{\mathrm{AB}}$$

The boundaries of atomic basins used for integration to determine each atomic energy contribution in the IQA method are defined by the zero‐flux condition of the ED gradient, as specified in QTAIMC [34].

The energetic characteristics of atomic interactions are evaluated based on the corresponding electrostatic and exchange pair contributions, which depend on the strength and type of interactions. However, the interaction energies between two atoms cannot serve as a universal predictor for the existence of a bond path between them [52]. Furthermore, these energies do not always correlate with interatomic distances or the values of ED at bond CPs. This is not paradoxical: strictly speaking, the ED within two interacting basins indeed depends on all atoms in a system.

Note that some contradictions are observed in the described approaches to chemical bonding. For instance, in QTAIMC, the necessary and sufficient condition for the existence of a chemical bond between atoms in an equilibrium state is the presence of bond paths (or BCP). The RDG analysis does not unambiguously confirm the presence of bond paths (or BCP) but instead identifies regions where either CP of the ED (RDG = 0) or low values of the ED and its reduced gradient are observed. In those regions, QTAIMC analysis does not detect bond paths between atoms; the ED gradient has a minimum in only two directions. This is due to the interatomic regions being influenced by overlapping boundaries of ED basins from neighboring atoms [41].

The characteristics of electron concentration and depletion in the position space of molecules and crystals can be determined through the analysis of isotropic internal local pressure within an inhomogeneous electronic continuum [53]. During the formation of chemical compounds, the electron cloud is self‐organized in the field of nuclei, creating regions with different electron concentration. In some areas, electron pressure is locally higher, while in others it is lower, with the resulting distribution depending on intra‐ and intermolecular interactions. The pressure distribution of the inhomogeneous ED in molecules and crystals is described by a symmetric macroscopic stress tensor of the second rank, $\sigma \left(r\right)=p\left(r\right)+\pi \left(r\right)$, which consists of a volumetric (isotropic) component $p\left(r\right)$ and a shear component $\pi \left(r\right)$.

Within the framework of density functional theory (DFT) [54], the stress tensor density, $\sigma \left(r\right)$, consists of an electrostatic component $\sigma_{\mathrm{ij}}^{M}\left(r\right)$ and quantum contributions, including the kinetic $\sigma_{\mathrm{ij}}^{S}\left(r\right)$ and exchange‐correlation $\sigma_{\mathrm{ij}}^{\mathrm{xc}}\left(r\right)$ parts:

(5) $$\sigma_{\mathrm{ij}}\left(r\right)=\sigma_{\mathrm{ij}}^{M}\left(r\right)+\sigma_{\mathrm{ij}}^{S}\left(r\right)+\sigma_{\mathrm{ij}}^{\mathrm{xc}}\left(r\right)$$

For hydrostatic stress distribution ($\pi \left(r\right)$ = 0), it is convenient to go to the scalar function

(6) $$p\left(r\right)=-\frac{1}{3}\mathrm{Tr}\;\sigma \left(r\right)$$

which presents a mean isotropic pressure at each point in the electronic continuum. It defines the virtual work performed during the compression of a volume element of the electronic continuum at point *r*, while preserving the shape of the volume element. The electrostatic (Maxwellian) part of the stress [55] has no characteristic features and may be added to the external (nuclei) electric field. In DFT, the quantum part of the internal pressure of an inhomogeneous electron continuum can be now calculated [56] as follows:

(7) $$p\left(r\right)=p^{S}\left(r\right)+p^{\mathrm{XC}}\left(r\right)$$

Here

(8) $$p^{S}\left(r\right)=\frac{2}{3}t_{s}\left(r\right)-\frac{1}{4}\nabla^{2}\rho \left(r\right)$$

is the quantum kinetic part of the internal electronic pressure, $t_{s}\left(r\right)$ is the kinetic energy density of noninteracting electrons. It can be expressed in orbital or an orbital‐free approximations [57] The part of pressure of an inhomogeneous electronic continuum, associated with exchange‐correlation effects, is given by [53]:

(9) $$p^{\mathrm{XC}}\left[r\right]=\rho \left(r\right)v_{\mathrm{xc}}\left(r\right)-e_{\mathrm{xc}}\left(r\right)+\frac{s}{3}\frac{\partial e_{\mathrm{xc}}\left(\rho \right)}{\partial s}$$

It depends on the exchange‐correlation volumetric energy density of electrons, $e_{\mathrm{xc}}\left(\rho \right)$: $E_{\mathrm{xc}}\left[\rho \right]=\int e_{\mathrm{xc}}\left(\rho \right)dr.$ The symbol s refers to the dimensionless RDG, which is a standard quantity used in the Generalized Gradient Approximation (GGA) $s\left(r\right)=\left|\nabla \rho \left(r\right)\right|/2k_{f}\left(r\right)\rho \left(r\right)$. It measures how quickly the ED *ρ*(**r**) changes in space (the inhomogeneity of the ED). The local Fermi wavevector is $k_{f}\left(r\right)={\left(3\pi^{2}\rho \left(r\right)\right)}^{\frac{1}{3}}$.

The quantum internal pressure of an inhomogeneous electron gas is thus associated with the kinetic part $p^{S}\left(r\right)$, the exchange part $p^{X}\left(r\right)$, and the correlation part $p^{C}\left(r\right)$, and it can be calculated [56] nonempirically as follows:

(10) $$p\left(r\right)=p^{S}\left(r\right)+p^{X}\left(r\right)+p^{C}\left(r\right)$$

We calculated the distribution of the isotropic internal pressure in the inhomogeneous electronic continuum (Equation 10). The kinetic energy density of noninteracting electrons, *t* ^*S*^(**r**), was approximated using the Kirzhnits expansion [58], which depends only on the ED *ρ*(**r**), its gradient, and its Laplacian. These quantities are well‐defined and can be derived from both theoretical wave functions and experimental X‐ray electron densities.

The exchange component of the pressure, *p* ^*X*^(**r**), was evaluated within the GGA using a standard exchange functional. The correlation part, *p* ^*С*^(**r**), was neglected as in previous works [56], under the assumption of its minor contribution. This methodology provides additional physical justification for the classification of the studied NCIs.

## 3 Computational Details

Geometry optimization and wavefunction calculations for the conformers of the neutral molecule and zwitterion were performed using the Kohn‐Sham method at the B3LYP/6‐311++G** level of theory [59, 60, 61] with the Gaussian 09 program. This computational level provides an accurate description of conformer geometries and relative energies [62]. Wavefunctions for zwitterionic conformers were calculated with implicit solvation effects modeled using the SMD method [63, 64].

Experimental ED data were derived from precision X‐ray diffraction measurements at 20 K for the molecular crystal of DL‐serine and were represented using parameters of the multipole model [65]. The structure was refined in space group P2_1_/a with excellent data quality, including *R*(*F*) ~1.5%–2.6% and residual ED below ±0.2 e/Å^3^. The geometry of the molecular cluster of seven serine molecules was taken from the crystal structure of DL‐serine. The cluster geometry was optimized using the Kohn‐Sham method at the B3LYP/6‐311++G(d,p) level. The absence of imaginary IR vibrational frequencies in the optimized structures of both the single molecule and the cluster was checked using the rigid rotor harmonic oscillator model [66]. The resulting multi‐electron wavefunctions were used to calculate theoretical ED and its characteristics.

The identification, classification, and quantitative description of NCIs were performed using QTAIMC theory [34], RDG analysis [38, 39, 40], and the IQA method. Calculations were carried out with the AIM2000 [67], Multiwfn [68], and AIMAll [69] programs.

The distribution of kinetic and exchange components of the quantum internal pressure in the inhomogeneous electronic continuum for the zwitterion and cluster was calculated from wavefunctions using Multiwfn. For the crystal, kinetic, exchange, electrostatic components, and total pressure were computed from experimental ED data using the WinXPRO program [70].

## 4 Computational Results

### 4.1 Neutral and Zwitterionic Serine Molecules

#### 4.1.1 Conformation Analysis

**FIGURE 2:**
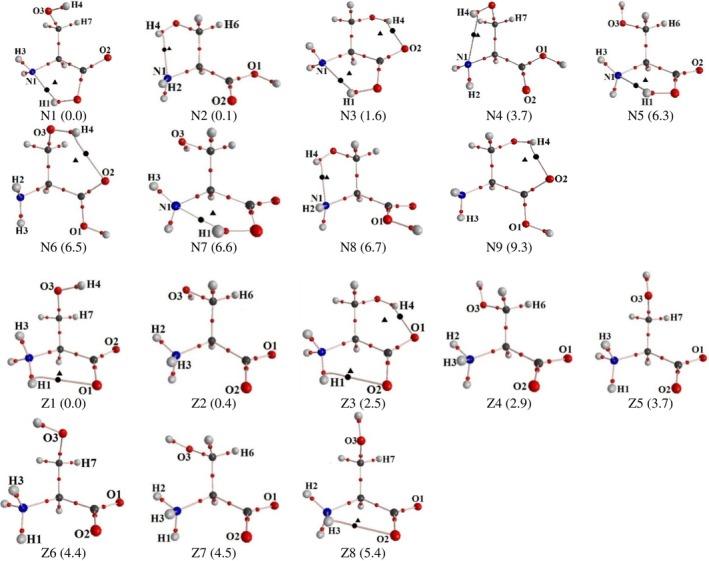
QTAIMC analysis of neutral and zwitterionic serine conformers. Conformers within 10 kJ/mol of the lowest‐energy structure are shown (Δ*E*_tot_ in kJ/mol is given in parentheses below each structure). Bond and ring CPs are shown as black dots and triangles, respectively.

The neutral serine molecule has five internal axes, rotation around which leads to the formation of various conformations (Figure 1). Analysis of the potential energy surface for the nonionized serine molecule, calculated as a function of the conformational state of the main chain, revealed six local minima (Figure S1). For each minimum, conformers with different conformational states of the side chain were identified, resulting in a total of 63 conformers (designated as N). The most stable conformers, with relative energies not exceeding 10 kJ/mol, are shown in Figure 2. Torsion angles and relative energies of these conformers determined through full geometry optimization (B3LYP/6‐311++G**), are provided in Table S1. The individuality of the found conformers/tautomers was confirmed by comparing geometries by distances between each atom and the centroid point [71, 72].

The geometric parameters of found conformers closely match those reported by other authors [73, 74, 75, 76]. The four most energetically stable conformers in Figure 2—N1, N2, N3, and N4—retain their positions in the stability hierarchy, with minor variations depending on the basis sets and computational methods used. These conformers have been observed experimentally [75, 76, 77, 78]. Conformers with relative energies exceeding 5 kJ/mol are present in negligible amounts [79], making their identification in experimental IR spectra challenging or even unfeasible. A comparison of the structures of the most stable conformations of serine and its derivative HCO‐NH‐CHCH_2_OH‐CONH_2_, with a longer main chain [80], demonstrated that the conformational properties of the amino acid align well with its structural organization as residues in peptides. However, the details of conformational stability in each system are naturally determined by the types of interactions, which differ between peptides and the acid.

Based on the calculated wavefunctions for the nine most stable conformers of neutral serine (N1–N9) with relative energies not exceeding 10 kJ/mol, the identification, classification, and quantitative description of intramolecular NCIs were performed within the QTAIMC framework. Relative energies, geometric parameters, and quantum‐topological characteristics of the bond CPs associated with the identified interactions are provided in Table S2. All intramolecular interactions in serine conformers can be categorized into the following groups based on the types of interacting molecular fragments: amino group of the main chain—carboxy group of the main chain, amino group of the main chain—hydroxyl group of the side chain, and carboxy group of the main chain—side chain.

Stable conformers of zwitterionic serine were obtained by optimizing the geometries of structures derived from previously identified neutral molecule conformers (63 structures). This was achieved by artificially transferring a hydrogen atom from the COOH group to the lone pair region of the NH_2_ group, with implicit solvation in water modeled using the SMD (water) method. After geometry optimization and vibrational frequency analysis, 13 unique zwitterionic conformers (designated as Z) were identified (Figure 2). Previously, conformational searches using MMFF and SYBYL force‐field methods for the L‐serine zwitterion identified only 9 conformers, which retained their individuality after optimization with B3LYP/6‐31+G(d,p), M06‐2X/6‐31+G(d,p), and MP2/6‐31+G(d,p) levels of theory, employing implicit solvation via the IEF‐PCM model [81]. Torsion angles and relative energies (Δ*E* ≤ 10 kJ/mol) for the zwitterionic serine conformers, obtained through full geometry optimization at the B3LYP/6‐311++G** level, are presented in the Supporting Information. The structures and stability ranking of the five most stable zwitterionic conformers agree with results from [82], based on optimization at the B3LYP/6‐31+G(d,p) level of theory. Experimental data (IR, Raman, FarIR) indicate the presence of the three most stable zwitterionic conformers in aqueous serine solutions at pH = 5.82 [82]. The geometries of the most stable conformers identified in this study are also consistent with results from [83], whose calculations at the B3LYP/6‐311++G(d,p) level and experimental vibrational absorption (VA) and vibrational circular dichroism (VCD) spectroscopy confirmed the presence of the three most stable conformers in neutral aqueous solutions.

#### 4.1.2 QTAIMC Analysis

QTAIMC analysis was performed for both neutral and zwitterionic conformers of serine to investigate the nature of covalent and NCIs. For the neutral forms, the analysis revealed the bond paths and BCPs corresponding to covalent bonds and structure‐stabilizing NCIs, primarily hydrogen bonds. Traditionally, a hydrogen bond is described as a three‐atom interaction with hydrogen at the center, following the D—H···A (donor–hydrogen···acceptor) notation. This convention is adopted in the present work to describe interactions that are accompanied by the formation of a bond path, as defined by QTAIMC. The most notable hydrogen bonding motifs include O1‐H1···N1 (observed in conformers N1, N3, N5, and N7), O3‐H4···N1 (N2, N4, and N8), and O3‐H4···O2 (N3, N6, and N9). These interactions contribute to the overall stabilization of the neutral serine conformers (Figure 2).

In contrast to the neutral molecule, where each conformer exhibits at least one hydrogen bond, such interactions are much less common in the zwitterionic form. QTAIMC analysis of the eight most stable zwitterionic conformers of serine (Z1–Z8, Δ*E*_tot_ ≤ 10 kJ/mol) revealed that hydrogen bonding in this form is primarily limited to N–H···O and O–H···O interactions. Notable examples include N1–H1···O1 (Z1), N1–H1···O2 (Z3), N1–H3···O2 (Z8), and O3–H4···O1 (Z3). The relative energies, quantum topological characteristics of CPs, and geometric parameters of these intramolecular interactions are summarized in Table S4. One might expect that the oppositely charged atomic groups within the main chain of the zwitterion would consistently attract each other and thus form stabilizing hydrogen bonds. However, this is rarely observed, which could be attributed to the use of the SMD (water) solvation model in the calculations.

Interestingly, the orientation of the side chain in conformer N1 closely resembles that in Z1, even though this arrangement is not enforced by hydrogen bonding. Nevertheless, it turns out to be energetically favorable, which is rather surprising. In both N3 and Z3, the side chain adopts the same orientation, stabilized by an identical hydrogen bond. As chemists, we would intuitively expect N3 and Z3 to be the most stable conformers by a significant margin, given that they each exhibit two intramolecular hydrogen bonds instead of one. Yet, this is not the case. We will attempt to address the reasons for this unexpected result in the following section.

#### 4.1.3 RDG Analysis

The RDG analysis was performed for both neutral and zwitterionic serine conformers to identify and characterize intramolecular interatomic regions with low ED values and RDG minima, which are indicative of potential NCIs.

**FIGURE 3:**
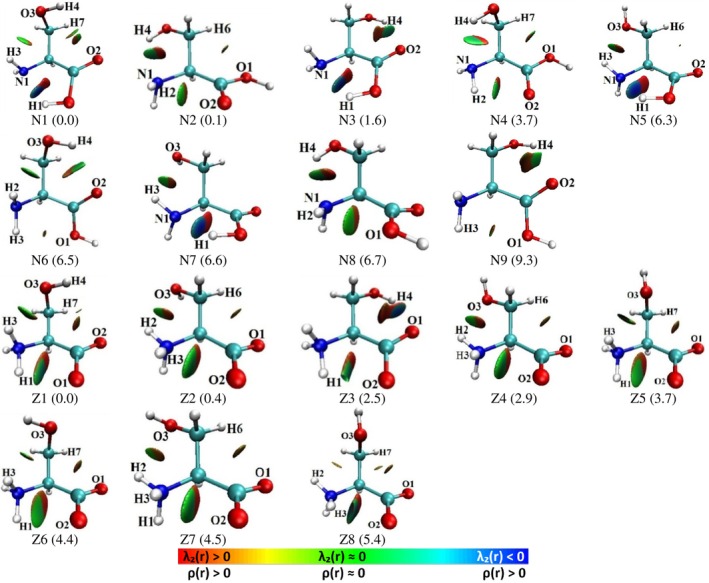
RDG isosurfaces plotted at 0.6 a.u. for selected neutral and zwitterionic serine conformers. Only conformers within 10 kJ/mol of the lowest energy structure are shown. The relative electronic energy difference (Δ*E*_tot_, in kJ/mol) is indicated in parentheses below each structure.

For neutral serine, such regions were observed between atoms separated by distances characteristic of NCIs (Figure 3). When a bond path exists within these regions, the RDG—sign[*λ*_2_(**r**)]*ρ*(**r**) diagram exhibits a spike that touches the x‐axis, indicating a zero ED gradient at the BCP and confirming a completed NCI. In contrast, when no bond path is present, RDG peaks are less pronounced (see Figure S2), suggesting the presence of *latent* intramolecular NCIs. In such cases, ED basin boundaries of neighboring atoms touch and penetrate the interatomic region, but the gradient minimum occurs in only two directions, preventing the formation of bond paths and BCPs [41].

The identified *latent* interactions in neutral serine conformers include H2···O1 (N8), H2···O2 (N2, N4), H2···O3 (N6), H3···O3 (N1, N5, N7), and H7···O1 (N4). Additionally, interatomic NCIs with distances exceeding the sum of van der Waals radii (2.600 Å for H···O and 2.650 Å for H···N) [30] were found: H6···O1 (N2), H6···O2 (N5), H7···O2 (N1), H3···O1 (N6, N9), and H4···O2 (N1). RDG analysis (Figures 3, and S2) further supports the classification of these NCIs as *latent* interactions.

For zwitterionic serine, RDG analysis (Figures 3 and S3) and interatomic distance measurements revealed a similar pattern of latent intramolecular NCIs. These include N1‐H1···O2 (Z5, Z6), N1‐H3···O2 (Z2, Z4, Z7), N1‐H2···O3 (Z2, Z4, Z7), and N1‐H3···O3 (Z1, Z5) (Figure 2). Additionally, interactions involving atom pairs with distances exceeding the van der Waals radii sum for H and O atoms were identified: N1‐H1···O2 (Z2, Z4, Z7, Z8), N1‐H2···O3 (Z8), N1‐H3···O1 (Z1), N1‐H3···O2 (Z3, Z5, Z6), N1‐H3···O3 (Z6), O3‐H4···O2 (Z1), C3‐H6···O1 (Z2, Z4, Z7), C3‐H7···O1 (Z5, Z6, Z8), and C3‐H7···O2 (Z1).

These findings help to understand the notable similarity between conformers N1 and Z1: both exhibit an almost identical pattern of latent interactions between the side chain and the backbone. This raises a fundamental question: could the presence of three weak, latent interactions provide an energetic stabilization comparable to (or even exceeding) that of an additional well‐defined hydrogen bond, as observed in conformers N3 and Z3?

To explore the nature of *latent* interactions and their role in molecular structuring, we analyzed the low‐frequency vibrational modes of the studied neutral and zwitterionic serine conformers.

**FIGURE 4:**
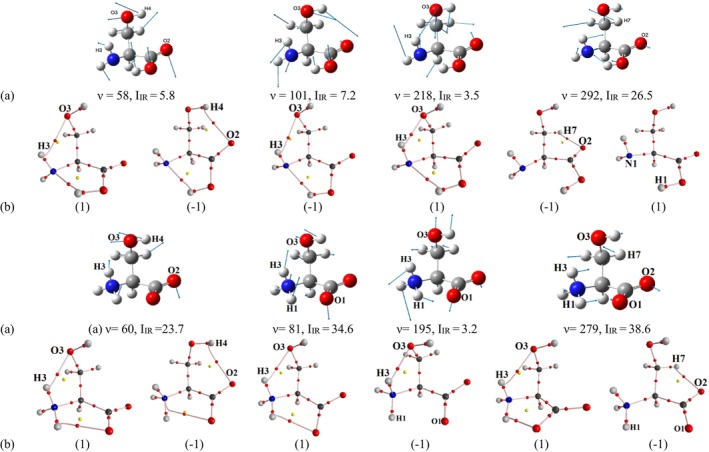
(a) Selected harmonic low‐frequency normal modes of conformer N1 of the neutral serine and Z1 of zwitterion. Arrows indicate the directions of atomic displacements, with the arrow length proportional to the displacement amplitude: *ν*—harmonic vibration frequency (cm^−1^), *I*_IR_—infrared intensity (km/mol). (b) Nonequilibrium structures. Atomic displacements from the start to the end of the vector are marked as (1), while displacements in the opposite direction are marked as (‐1). For *ν* = 60 cm^−1^ (‐1), see more Figure S10.

For neutral serine, atoms involved in both *completed* and *incomplete latent* NCIs exhibited significant vibrational amplitudes (Figure 4). It is reasonable to assume that atomic displacements during vibrations could lead to the formation and rupture of interatomic ED bridges (“pseudo‐bond paths”), which locally reduce the potential energy of electrons. To test this hypothesis, we examined interatomic distances and ED distribution in nonequilibrium structures for the H3···O3, H4···O2, and H7···O2 atom pairs in the most stable conformer N1, as they were previously classified as *latent* interactions.

The H3···O3 bridge appears in nonequilibrium structures (Figure 4) at vibrational frequencies of 58 cm^−1^ (forward), 218 cm^−1^ (forward), and 101 cm^−1^ (backward), depending on the direction of atomic displacement. This vibrational behavior supports the classification of H3∴O3 as a *dynamic latent* interaction; we denote it as (∴) to reflect the consistent formation of a pseudo‐bond path during atomic displacements.

In contrast, for H4···O2, an ED bridge appeared at only one vibrational frequency (58 cm^−1^, forward) when atoms moved toward each other, suggesting that H4··O2 is a *static latent* (*less dynamic*) interaction, unlikely to participate in a “pulsating” network of atomic contacts. Accordingly, we denote it with two dots below (··). Similarly, the H7··O2 bridge emerged at 292 cm^−1^ (forward) when atoms moved toward each other.

Interestingly, the bond path of the *completed* H1···N1 interaction remained almost intact at all examined frequencies (58, 101, 218, 292, 344, and 367 cm^−1^), except for two cases at 292 and 367 cm^−1^, where it temporarily vanished in nonequilibrium structures.

A similar vibrational analysis was conducted for all latent interactions identified using RDG (Table S3). The following interactions exhibited clear pseudo‐bond path formation and can be classified as *dynamic latent* interactions: H7∴O1 (N4), H2∴O2 (N4), H3∴O3 (N5), H2∴O3 (N6), H3∴O3 (N7), H2∴O1 (N8). In contrast, the interactions H2··O2 (N2), H6··O1 (N2), H6··O2 (N5), H3··O1 (N6), H3··O1 (N9) did not exhibit stable pseudo‐bond formation and can therefore be classified as *static latent* interactions.

As in the neutral molecule, atomic displacements along vibrational modes in zwitterionic serine led to the formation of pseudo‐bond paths for several *latent* interactions. In the most stable conformer Z1 (Table S5, Figure 4b), the *dynamic* H3∴O3 interaction exhibited bridges at 60, 81, and 297 cm^−1^ (forward), and at 195 cm^−1^ (backward), depending on the direction of atomic movement. For the more *static* H7··O2 and H4··O2 interactions, bridges appeared at 279 cm^−1^ and 60 cm^−1^ (forward), respectively, under similar conditions.

At frequencies 195 and 279 cm^−1^, the completed N1‐H1···O1 bond path disappeared, similar to what was observed in neutral serine. Notably, at 60 cm^−1^, a new N1···O1 pseudo‐bond path emerged, replacing the original bond path of H1···O1. This behavior was also observed in Z4 at 198.6 cm^−1^ and in Z7 at 323 and 407 cm^−1^. For most *completed* NCIs, such as N1‐H1···O1 (*E*_int_ = −413.5 kJ/mol), the bond path was preserved at all vibrational frequencies except 334 cm^−1^.

A systematic vibrational analysis of all *latent* interactions in zwitterionic serine identified several cases where pseudo‐bond path formation was consistently observed. The interactions H2∴O3 (Z2, Z4, Z7), H3∴O2 (Z2, Z4, Z7), H1∴O2 (Z5, Z6), and H3∴O3 (Z5) were classified as *dynamic latent* interactions, as they showed clear pseudo‐bond path formation in nonequilibrium structures. Meanwhile, interactions such as H1··O2 (Z2, Z4), H6··O1 (Z2, Z4, Z7), H3··O2 (Z3, Z5, Z6), H7··O2 (Z5, Z6, Z8), H3··O3 (Z6), H1··O2 (Z7, Z8), and H2··O3 (Z8) were classified as *static latent* interactions, as no consistent pseudo‐bond formation was observed during vibrational analysis.

In summary, vibrational analysis supports the classification of certain *latent* interactions as *dynamic*, meaning they contribute to a “pulsating” network of atomic contacts in which pseudo‐bond paths can dynamically form or break. These *dynamic* interactions significantly influence the structural organization of serine, particularly in its zwitterionic form, where the interplay of electrostatic and vibrational effects creates a unique environment for NCIs.

#### 4.1.4 IQA Analysis

To further investigate the nature of completed and latent NCIs identified via RDG, the IQA method was used to calculate the pairwise interatomic interaction energies (*E*_int_) in both neutral and zwitterionic serine conformers.

**FIGURE 5:**
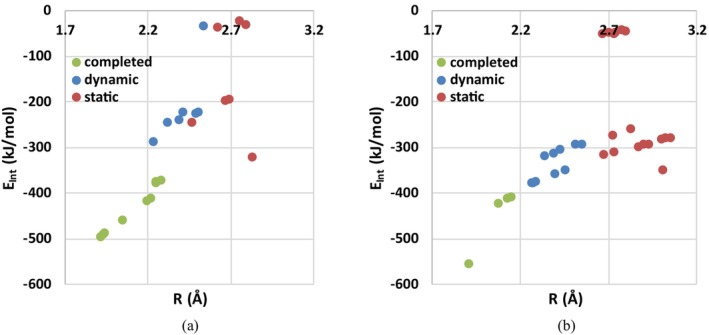
Interaction energy *E*_int_ (in kJ/mol) versus distance *R* (in Å) for NCIs in studied serine conformers: (a) Neutral conformers and (b) Zwitterionic conformers.

In the neutral serine conformers, the *E*_int_ values for all identified interatomic NCIs were negative, indicating that these interactions contribute to system stabilization [84] (Figure 5a). However, not all NCIs correspond to bond paths, meaning they do not necessarily have a structure‐forming function. For *completed* (structure‐forming) NCIs, such as O1‐H1···N1 (N1, N3, N5, and N7), O3‐H4···N1 (N2, N4, and N8), and O3‐H4···O2 (N3, N6, and N9), the *E*_int_ values were significantly more negative compared to other atom pairs (Tables S2 and S3).

Although no direct correlation between interaction energy (*E*_int_) and interatomic distances was identified (since in addition to the distance there is also the influence of the surrounding atoms on the resulting *E*_int_), it can be observed that shorter distances often correspond to more favorable (i.e., more negative) interaction energies. However, this trend is not always easy to observe or clearly confirm. This distance dependence can be seen for both *completed* and *latent* interactions, including H2···O1(2), H3···O1(O3), H2···O3, and H4···O2. Notably, in the case of C–H···O interactions, *E*_int_ values were more negative for atom pairs involving the carbonyl oxygen O2 (H···O2 distances: 2.619–2.789 Å) compared to those involving the hydroxyl oxygen O1 (H···O1 distances: 2.537–2.751 Å) (Table S3).

Electrostatic interactions contribute significantly to the binding energy of all NCIs. However, no strict correlation was observed between the electrostatic contribution and the nature of the interaction (*completed* vs. *latent*). Transitioning from O‐H···N and O‐H···O hydrogen bonds (*completed*) to N‐H···O interactions (*latent*) led to a slight increase in the electrostatic contribution (~92.1% → ~95.2%). In contrast, for C‐H···O interactions (*latent*), the electrostatic contribution decreased to ~70.6% (Tables S2 and S3).

To further distinguish between interacting and noninteracting atoms, we analyzed changes in intra‐atomic energies ($\Delta E_{\text{intra}}$), which reflect the internal response of an atom to its environment and can be linked to charge redistribution effects. For instance, in conformer N1, the H3∴O3 pair was classified as a dynamic latent interaction: $E_{\mathrm{int}}$ < 0, with a slight destabilization of H3 ($\Delta E_{\text{intra}}$(H3) = +1.6 kJ/mol) and significant stabilization of O3 ($\Delta E_{\text{intra}}$(O3) = −17.7 kJ/mol). According to the established interpretation (e.g., Ayers, Bultinck, Martín Pendás), such trends are indicative of partial charge transfer: in this case, O3 gains ED and becomes more stabilized, while H3 loses ED and is destabilized.

In conformer N3, this interaction vanishes due to side‐chain rotation, and the corresponding $E_{\text{intra}}$ values change accordingly. A similar analysis of the H4··O2 and H7··O2 pairs supports their classification as latent interactions: they persist across conformers, despite relatively weak stabilization effects (Table S3), again suggesting subtle but consistent ED redistribution rather than strong bonding.

For zwitterionic serine conformers, the IQA analysis showed a similar stabilization trend, but with a greater contribution from charge‐assisted hydrogen bonding (Figure 5b). The interaction energies for *completed* (structure‐forming) hydrogen bonds H1···O1, H1···O2, H3···O2, and H4···O1 are highly negative, with values ranging from −408.9 to −556.3 kJ/mol (Table S5).

The electrostatic contribution remained dominant across all interaction types. However, as in neutral conformers, no direct correlation was observed between electrostatic energy and interaction type. The exchange energy contributions ($V_{x}^{\mathrm{AB}}$) varied: 6.5%–8.6% for *completed* N‐H···O and O‐H···O hydrogen bonds, 0.4%–4.9% for *latent* N‐H···O and O‐H···O interactions, 18.0%–22.5% for *latent* C‐H···O interactions.

A key consideration for the zwitterionic form was the symmetry of the NH_3_^+^ group during its rotation and subsequent optimization, as it affects the numbering of hydrogen atoms. In conformer Z3, the H2 and H3 atoms in NH_3_^+^ were equivalent due to threefold rotational symmetry. A noninteracting reference hydrogen atom H2 in Z3 was selected for comparison across conformers.

Atomic energy analysis further confirmed that NCI such as H3∴O3, H4··O2, and H7··O2 in conformer Z1 could be classified as *latent* interactions. For instance, the H4··O2 pair resulted in slight stabilization of H4 ($\Delta E_{\text{intra}}^{H4}$ = −34.9 kJ/mol) and O2 (${\Delta E}_{\text{intra}}^{O2}$ = −20.1 kJ/mol) compared to Z4, where this interaction disappeared due to side‐chain rotation. In contrast, the H7···O2 pair in Z1 exhibited H7 destabilization ($\Delta E_{\text{intra}}^{H7}$ = +7.0 kJ/mol) and O2 stabilization ($\Delta E_{\text{intra}}^{O2}$ = −20.1 kJ/mol) compared to the noninteracting reference state in Z4. These variations in intra‐atomic energies are consistent with localized charge redistribution effects, suggesting partial charge transfer between the interacting atoms.

A full atomic energy analysis was conducted for all zwitterionic conformers (Table S5), confirming the classification of the following *latent* interactions: H1···O2 (Z2, Z4, Z5, Z6, Z7, Z8), H7···O2 (Z1), H7···O1 (Z6), H6···O1 (Z2), H7···O1 (Z5, Z8), where the H atom is stabilized and the O atom destabilized; and H7···O1 (Z6), H6···O1 (Z7), where both the H and O atoms are destabilized.

While $E_{\mathrm{int}}$ values show a clear trend, being most negative for completed interactions and less negative for latent ones, this trend is primarily governed by the Coulombic component (*E*_cl_), which contributes over 90% of the total interaction energy. However, we acknowledge that *E*_cl_ is dominated by monopolar terms at long distances and may not reliably indicate bonding. Therefore, we no longer interpret the presence of non‐covalent bonds based solely on *E*_cl_. Notably, in the neutral form of serine, the exchange component ($V_{x}^{\mathrm{AB}}$) becomes positive for latent interactions, unlike in the zwitterionic form, where it remains negative but decreases in magnitude from completed to *latent* NCIs. (Figure S22).

#### 4.1.5 Local Pressure Analysis

**FIGURE 6:**
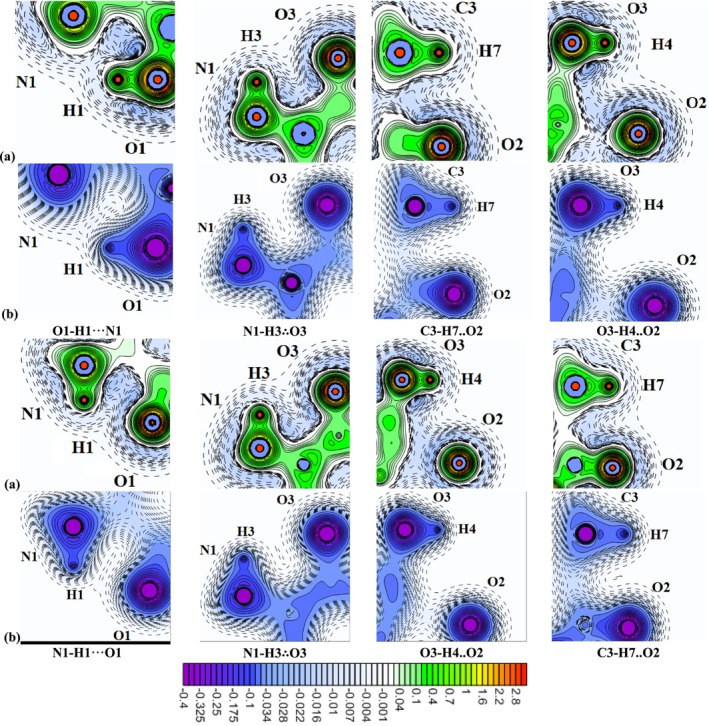
Distribution of the kinetic (a) and exchange (b) components of internal electronic pressure in the N1 conformer of the neutral serine molecule and Z1 conformer of serine zwitterion; cross‐sectional planes are taken through the specified atoms. Dashed lines represent values ranging from −0.034 to −0.010 a.u. with increments of 0.002 a.u.; from −0.010 to 0 a.u. with increments of 0.001 a.u. Solid lines represent values ranging from −0.400 to −0.050 a.u. with increments of 0.025 a.u.; from 0 to 0.1 a.u. with increments of 0.002 a.u.; from 0.1 to 1.0 a.u. with increments of 0.1 a.u.; and from 1.0 to 3.0 a.u. with increments of 0.2 a.u.

The kinetic and exchange components of internal electronic pressure provide valuable insights into the nature of covalent and NCIs in serine conformers. These functions for the N1 conformer of neutral serine are illustrated in Figure 6. The electronic continuum in the kinetic component maps is compressed along the *covalent* N1‐H3, C3‐H7, O3‐H4, and O1‐H1 bonds, forming local bridges corresponding to the electron concentrations of bonding electron pairs. These bridges are separated by saddle points in the kinetic pressure function *p* ^*S*^(**r**), which are shifted along bond paths toward the more electronegative atom, away from the BCPs of the ED (Figure 6a). Maxima in *p* ^*S*^(**r**), corresponding to the nonbonding (lone) electron pairs of the N1, O3, O2, and O1 atoms, are also observed. Their spatial arrangement follows the tetrahedral geometry of N1, O3, and O2, characteristic of single bonds, and the planar geometry of O1, typical of a carbonyl group. The kinetic contribution of electrons to pressure significantly influences the localization of electron pairs near these atoms (Figure 6a). The distribution of the exchange component *p* ^*X*^(**r**) follows a similar pattern, being associated with saddle points of the ED along covalent bond paths and lone electron pairs, corresponding to minima in *p* ^*X*^(**r**).

For the completed H1···N1 interaction in the N1 conformer, a region of negative *p* ^*S*^(**r**) values appears between the atoms, while a local minimum in *p* ^*X*^(**r**) coincides with the BCP (Figure 6). A similar distribution of *p* ^*S*^(**r**) is observed between atoms H3/O3 and H7/O2, which were classified as *dynamic latent* interactions. However, for these interactions, the local minima in *p* ^*X*^(**r**) are positioned closer to the molecular backbone and shifted away from the interatomic line (Figure 6b). In contrast, for the H4···O2 air, which was classified as a static latent interaction, the kinetic component *p* ^*S*^(**r**) exhibits significantly less negative values. Additionally, in the exchange pressure distribution, either a local minimum is absent, or it becomes significantly shallower (less negative than −0.001 a.u.). This suggests that the exchange component of internal electronic pressure provides a detailed characterization of NCIs, particularly in cases involving electron delocalization.

In all studied conformers, the kinetic contribution of electrons to the internal pressure confirmed the three‐dimensional organization of bonding and lone electron pairs. Meanwhile, the exchange component plays a dominant role in determining electron distribution in interatomic regions. All *completed* NCIs, including O1‐H1···N1 (N1, N3, N5, N7), O3‐H4···N1 (N2, N4, N8), and O3‐H4···O2 (N3, N6, N9), are characterized by negative *p* ^*S*^(**r**) values in interatomic space and the presence of local minima in *p* ^*X*^(**r**), coinciding with BCPs (Figure S7).

*Dynamic latent* interactions, such as N1‐H2∴O2 (N4), N1‐H3∴O3 (N1, N5, N7), and N1‐H2∴O3 (N6), exhibit a similar pressure distribution to conformer N1, with negative *p* ^*S*^(**r**) values in the interatomic space and local minima in *p* ^*X*^(**r**) located away from the internuclear line (Figure S8). However, for *static latent* interactions such as N1‐H3··O1 (N9) and C3‐H6··O1 (N2), this structural feature is either absent or the corresponding minima become significantly less pronounced (below 0.001 a.u.).

For fragments where H…O pair were classified as forming latent interactions, the kinetic component p^S^(**r**) in the interatomic space remains negative. However, in interactions such as N1‐H2··O2 (N2), N1‐H2∴O1 (N8), and C3‐H7∴O1 (N4), the *p* ^*X*^(**r**) function exhibits unfinished minima, which become shallower and shift deeper into pseudo‐cycles as the H…O interatomic distance increases (2.460, 2.410, and 2.530 Å, respectively). In contrast, for interactions N1‐H3··O1 (N6), O3‐H4··O2 (N1), and C3‐H6··O2 (N5), such structural features are either absent or significantly less pronounced (Figure S9).

Thus, the exchange component of electronic pressure for all *latent* interactions exhibits local minima in *p* ^*X*^(**r**), which are most pronounced in *dynamic latent* interactions. In contrast, for *static latent* interactions, minima of the exchange pressure function are not observed in all studied conformers. Even when present, they remain incomplete and shift deeper into pseudo‐cycles as the interatomic H…O distance increases.

The analysis of internal electronic pressure, particularly the distribution patterns of the kinetic *p* ^*S*^(**r**) and exchange *p* ^*X*^(**r**) contributions, provides further insights into the nature of NCIs. In neutral serine, *dynamic latent* interactions were characterized by the formation of interatomic regions with negative *p* ^*S*^(**r**) and *p* ^*X*^(**r**) values, accompanied by local minima in *p* ^*X*^(**r**). In contrast, *static latent* interactions lacked these features, likely due to increased exchange pressure values in the interatomic region (Figure S13).

For the Z1 conformer, these functions are shown in Figure 6. The electronic continuum is compressed along the covalent N1‐H1, N1‐H3, C3‐H7, and O3‐H4 bonds, forming local bridges corresponding to bonding electron pairs. These bridges are separated from BCPs along bond paths by saddle points in the kinetic pressure function *p* ^*S*^(**r**) (Figure 6). Maxima in *p* ^*S*^(**r**) are also observed, corresponding to the lone electron pairs of O3, O2, and O1, with spatial arrangements consistent with the tetrahedral geometry of hydroxyl oxygen O3 and the planar geometry of the carbonyl oxygens O1 and O2.

The *completed* H1···O1 interaction and the *dynamic latent* H3∴O3 interaction in Z1 are accompanied by the formation of regions with negative *p* ^*S*^(**r**) values and local minima in *p* ^*X*^(**r**) (Figure 6). For H1···O1, the local minima are more pronounced and coincide with the BCP, whereas for H3∴O3, they are shifted away from the internuclear line (Figure 6b). In contrast, for the H4··O2 and H7··O2 pairs, classified as static latent interactions, the kinetic component *p* ^*S*^(**r**) is significantly less negative, and in the exchange pressure distribution, the local minimum is either absent or significantly less pronounced.

In all studied conformers, the kinetic contribution of electrons to pressure confirmed the organization of bonding and lone electron pairs. The exchange component appears primarily responsible for electron distribution in interatomic space. All completed NCIs N1‐H1···O1 (Z1), N1‐H1···O2 (Z3), N1‐H3···O2 (Z8), and O3‐H4···O1 (Z3) exhibit negative *p* ^*S*^(**r**) values and local minima in *p* ^*X*^(**r**) at BCPs (Figure S11).

*Dynamic latent* interactions, such as N1‐H1∴O2 (Z5), N1‐H2∴O3 (Z2, Z4, Z7), N1‐H3∴O2 (Z2, Z4, Z7), and N1‐H3∴O3 (Z1), exhibit similar patterns with interatomic distances of 2.265 to 3.024 Å, except for N1‐H2∴O3 (Z7) (R(H···Y) = 2.923 Å) (Figure S12). In contrast, for *static latent* interactions, such as O3‐H4··O2 (Z1), N1‐H1··O2 (Z2, Z4, Z7, Z8), N1‐H2··O3 (Z8), N1‐H3··O2 (Z5, Z6), N1‐H3··O1 (Z1), C3‐H6··O1 (Z4, Z7), and C3‐H7··O1 (Z5, Z6, Z8), structural features of exchange pressure are either absent or significantly less pronounced (Figure S14). These findings confirm that *dynamic latent* interactions exhibit stronger *p* ^*X*^(**r**) minima, shorter interatomic distances, and larger D‐H…A angles, whereas *static latent* interactions lack these characteristics.

### 4.2 Comparison of Zwitterionic Serine With Molecular Cluster and Crystal

The comparison of the conformational state of the individual zwitterion Z6, the central zwitterion in a cluster of 7 molecules extracted from the crystal structure, and the zwitterion in the DL‐serine crystal revealed that their geometric parameters are similar (Table 1). Therefore, these systems were selected to trace the influence of the environmental effect as the individual zwitterion of serine transitions to the cluster and subsequently to the crystal.

**TABLE 1:** Torsional angles of the Z6 conformer, the central molecule in the 7‐molecule 7Z6 cluster, the DL‐serine molecule in the crystal at 20 K (experimental), and the periodic crystal structure (calculated).

Object	*φ* ^a^	*ψ* ^a^	*χ*_1_ ^a^	*χ*_2_ ^a^
Z6	−171.4	177.4	69.4	−90.0
7Z6^b^	−178.3	−178.4	69.2	−81.8
Cryst. exp.	−172.7	−178.8	68.7	−91.5
Cryst. calc.	−171.1	178.9	65.4	−88.0

^a^ Torsional angles: *φ* = (Н2‐N1‐C2‐C1); *ψ* = (N1‐C2‐C1‐O1); *χ*_1_ = (N1‐С2‐C3‐O3); *χ*_2_ = (С2‐С3‐О3‐Н4).

^b^ In the case of the 7Z6 cluster, the values are shown only for the central molecule.

**FIGURE 7:**
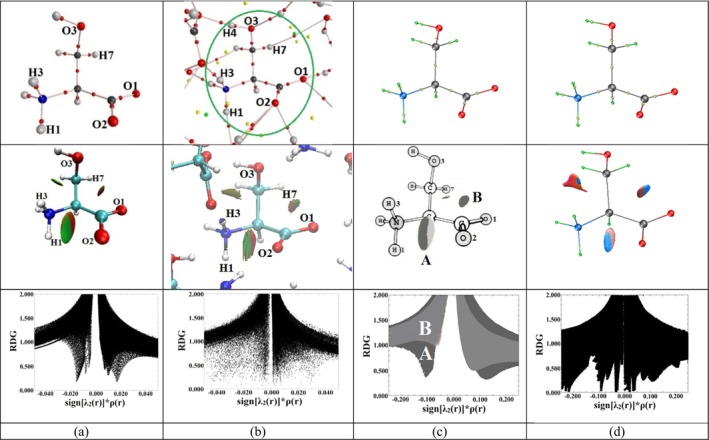
Analysis of noncovalent interactions in serine systems. (a) Isolated zwitterionic serine Z6; (b) Serine molecule within a 7‐molecule cluster (Z7G) extracted from the experimental [65] DL‐serine unit cell and optimized at the B3LYP/6‐311++G(d,p) level; (c) Serine molecule in the experimental [65] DL‐serine crystal at 20 K; (d) Periodic structure of crystalline DL‐serine optimized at the B3LYP/POB‐TZVP level. Top row: QTAIMC analysis showing atomic positions, bond paths, and CPs (atoms, bond, ring, and cage). Middle row: RDG isosurfaces (RDG = 0.6 a.u.) revealing noncovalent interactions, including latent intramolecular NCIs. Key interactions are marked as regions A and B. Bottom row: RDG versus sign(*λ*_2_)*ρ*(*r*) plots highlighting attractive (A) and repulsive (B) interactions.

QTAIMC analysis of the individual zwitterion conformer Z6, the zwitterion in a molecular cluster, and in a crystal has revealed the bond path network associated with covalent bonds within and between the molecules (Figure 7). Analysis of RDG in these systems revealed regions with low ED and small RDG values between the atomic pairs H1∴O2, H3··O2, H3··O3, and O1··H7 within the molecule in conformer Z6 and the cluster (Figure 7a,b). These regions do not correspond to bond paths in the QTAIMC framework. For the serine molecule in the crystal, analysis of the experimental ED distribution and its reduced gradient indicates that such regions are identified within the molecule between the atomic pairs H1···O2, H3···O2, and H7···O1 (Figure 7c). Just like in the isolated molecule and the 7Z6 cluster, the same pattern of noncovalent interactions is observed in the theoretically optimized crystal. (Figures 7d and S4) In the RDG region between H3 and O3, intermolecular interaction slightly contributes as well, even adding a bit more volume to the isosurface.

Thus, in the studied systems, the *latent* NCIs N1–H1∴O2, N1–H3··O2, and C3–H7··O1 interactions are consistently identified, while the appearance or disappearance of the N1–H3··O3 interaction is associated with the redistribution of ED upon the transition of the molecule into the crystal and the formation of intermolecular crystal‐stabilizing hydrogen bonds.

The depth of the sharp RDG peaks (“spikes”) (in Figure 7a–c) decreases with the reduction of sign(*λ*_2_)*ρ*. In this case, the ED decreases in the interatomic space as the distances between atoms increase in the sequence H1∴O2, H3··O2, H3··O3, and H7··O1 (e.g., for the individual zwitterion Z6, these distances are 2.453, 2.669, 2.721, and 2.726 Å, respectively, as shown in Figure 7a), indicating a weakening of these *latent* NCIs. The weakening of these uncompleted NCIs in the series N1‐H1∴O2, N1‐H3··O2, N1‐H3··O3, and C3‐H7··O1 in the Z6 zwitterion is also evidenced by the decrease in the pairwise interatomic interaction energies (*E*_int_), which are −351.1, −317.3, −274.9, and −51.8 kJ/mol, respectively. The primary contribution to these values comes from the classical electrostatic interaction energy. The exchange energy contribution for N1‐H1∴O2, N1‐H3··O2, and N1‐H3··O3 interactions ranges from 1.1% to 2.5% and increases for C3‐H7··O1 to 18.7% (Table 2). The fact that the RDG diagram for the Z6 conformer shows only three spikes (despite four possible interactions identified through 3D RDG analysis) is likely due to the overlap of two spikes corresponding to interactions with similar ED values in the interatomic spaces.

**TABLE 2:** Values of *latent* interatomic interaction energy *E*_int_ and their exchange V_X_^AB^ and electrostatic *E*_els_^AB^ components (kJ/mol), distance between atoms R(H···Y) (Å), and angle theta Θ(X‐H···Y) (degrees) in the Z6 zwitterion conformer, the central molecule in the cluster, and the crystal.

Fragment	Object	BCP	*E*_els_^AB^	V_X_^AB^	*E*_int_	R(H···Y)	Θ
N1‐H1∴O2	Z6	—	−342.5	−8.7	−351.1	2.453	89.4
	7Z6	—	−311.7	−8.3	−319.9	2.541	83.9
	Cryst. exp.	—	—	—	—	2.465	89.4
	Cryst. calc.	—	—	—	—	2.530	86.6
N1‐H3··O2	Z6	—	−313.8	−3.6	−317.3	2.669	77.7
	7Z6	—	−327.7	−7.1	−334.8	2.575	81.8
	Cryst. exp	—	—	—	—	2.667	77.8
	Cryst. calc.	—	—	—	—	2.738	75.7
N1‐H3··O3	Z6	—	−270.3	−4.6	−274.9	2.721	98.1
	7Z6	—	−284.6	−3.2	−287.8	2.871	90.2
	Cryst. exp	—	—	—	—	2.759	95.2
	Cryst. calc.	—	—	—	—	2.712	95.3
C3‐H7··O1	Z6	—	−42.1	−9.7	−51.8	2.726	91.9
	7Z6	—	−14.5	−18.7	−33.2	2.705	92.3
	Cryst. exp	—	—	—	—	2.672	92.4
	Cryst. calc.	—	—	—	—	2.642	94.5

**FIGURE 8:**
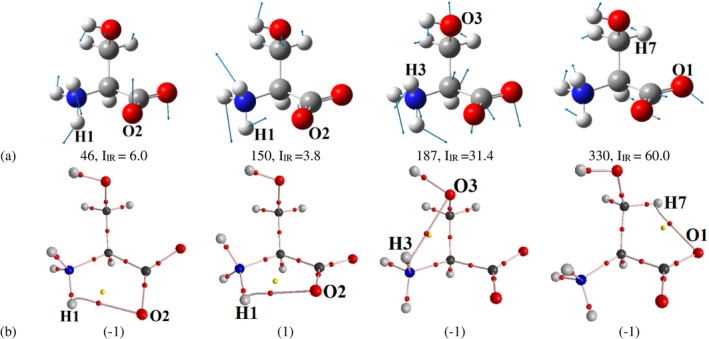
(a) Equilibrium structure of the serine zwitterion Z6 conformer with atomic displacement vectors corresponding to specific vibrational frequencies. (b) Nonequilibrium structures of the Z6 conformer. Atomic displacements along the vectors are labeled as (1), while displacements in the opposite direction are labeled as (‐1).

We conducted an analysis of interatomic distances and characteristics of the ED distribution in nonequilibrium structures for *latent* interactions H1∴O2, H3··O2, H3··O3, and H7··O1 in the Z6 conformer, whose geometry closely resembles that in the molecular crystal of DL‐serine. Quantum‐topological analysis of the ED for the atom pairs H1∴O2 and H7··O1 revealed that in the nonequilibrium structures of the Z6 conformer, formed during atomic vibrations, pseudo‐bond paths emerge (Figure 8b). For the H1···O2 contact, this is observed at vibrational frequencies *ν* = 46 (Figure 8b) when atoms are displaced opposite to the vectors shown in Figure 8a, and at *ν* = 150 cm^−1^ when displaced along the vectors. For the H7··O1 pair, an ED bridge forms between the atoms at *ν* = 330 cm^−1^ (Figure 8b) when atoms are displaced toward each other (opposite to the direction of vectors in Figure 8a).

Between H3 and O3, at *ν* = 187 cm^−1^ (Figure 8b), a pseudo‐bond bridge appears during atomic displacements toward each other (opposite to the vectors in Figure 8a), but it bypasses atom H3, curving around it to connect the nuclei of atoms N1 and O3. To rule out an error potentially caused by insufficiently precise standard settings in the AIM2000 program, the ED distribution and its gradient lines were recalculated in the plane of atoms N1, H3, and O3 (Figure S15), which clearly show the absence of pseudo‐bond paths for both H3…O3 and N1…O3. Thus, the NCIs between H3 and O2, H3 and O3, and H7 and O1 can be classified as *static latent* interactions. The likelihood of these atomic pairs participating in a “pulsating” network of atomic contacts during favorable vibrational patterns is low, as the appearance of a pseudo‐bond path is either not observed or observed at only one or two frequencies. For a more detailed analysis of NCIs in the Z6 conformer, in the central molecule within the cluster, and in the crystal, the kinetic *p* ^*S*^(**r**) and exchange *p* ^*X*^(**r**) contributions to the electron continuum pressure were calculated (Figures S16 and S17). Additionally, for the molecule in the crystal, the electrostatic contribution and the total electronic pressure were computed (Figure S17). Nonetheless, we anticipate that the presence of strong intermolecular hydrogen bonds in the calculated cluster and crystal may restrict the vibrational freedom necessary for the formation of intramolecular “pseudopaths” (Figures S5 and S6). As a result, an interaction previously classified as *dynamic* in the isolated molecule may shift toward a *static* character within the environment.

When the molecule transitions to a cluster, there is no clear correlation between *E*_int_ values (−319.9, −334.8, −287.8, −33.2 kJ/mol) and interatomic distances H‐O for the interactions N1‐H1∴O2, N1‐H3··O2, N1‐H3··O3, and C3‐H7··O1 (2.541, 2.575, 2.871, 2.705 Å, respectively) (Table 2). The exchange energy contribution to these interactions is similar to that observed in the isolated zwitterion molecule (1.1%–2.6%) and increases to 56.3% only for C3‐H7··O1. Comparing N1‐H1∴O2 in the isolated zwitterion and the cluster shows that increasing the H‐O distance makes *E*_int_ less negative (conformer Z6: *E*_int_ = −351.1 kJ/mol, H‐O distance = 2.453 Å; cluster conformer: *E*_int_ = −319.9 kJ/mol, H‐O distance = 2.541 Å). A similar trend is observed for N1‐H3··O2 (conformer Z6: *E*_int_ = −317.3 kJ/mol, H‐O distance = 2.669 Å; cluster conformer: *E*_int_ = −334.8 kJ/mol, H‐O distance = 2.575 Å). However, for N1‐H3··O3, when Z6 transitions to the cluster, the H‐O distance increases from 2.721 to 2.871 Å, but *E*_int_ becomes more negative (−274.9 to −287.8 kJ/mol). Thus, when the isolated zwitterion Z6 transitions to the cluster, the correlation between *E*_int_ values and H‐O distances for these *latent* intramolecular N‐H···O interactions weakens, likely due to environmental effects. The formation of completed intermolecular interactions and redistribution of ED between donor and acceptor atoms also plays a role. For instance, atoms H3 and O3 in the central molecule form hydrogen‐bond bridges with neighboring molecules, facilitating ED redistribution. Changes in the interaction C3‐H7··O1 in conformer Z6 are also notable: in Z6, the H‐O distance is 2.726 Å, and *E*_int_ equals −51.8 kJ/mol. In the cluster, the H‐O distance decreases to 2.705 Å, but the interatomic interaction energy *E*_int_ becomes less negative, reaching −33.2 kJ/mol. Calculating the energies of the specified pairwise interatomic interactions in the DL‐serine crystal using the IQA method is not feasible based on X‐ray diffraction experimental data since the calculations require single‐electron spin‐orbitals, which cannot be derived from the ED. Moreover, current IQA implementations do not yet support periodic wavefunctions.

These functions for the Z6 conformer of the serine molecule are shown in Figure S16. The electronic continuum is compressed along the covalent N1‐H1, N1‐H3, and C3‐H7 bonds, forming local bridges that correspond to the electron concentrations of bonding electron pairs. These bridges are separated from BCPs by saddle points in the kinetic component of the internal electronic pressure, *p* ^*S*^(**r**), which shifts toward the most electronegative atom along the bond paths (Figure S16). Maxima in *p* ^*S*^(**r**), corresponding to nonbonding (lone) electron pairs of atoms O3, O2, and O1, are also observed. The spatial arrangement of these maxima is consistent with the tetrahedral coordination of the hydroxyl oxygen O3 and the planar coordination of the carbonyl oxygens O1 and O2. The kinetic contribution of electrons to pressure significantly influences the localization of electron pairs near O3, O2, and O1 (Figure S16). The distribution of the exchange component, *p* ^*X*^(**r**), is also clearly associated with saddle points in the electronic density along covalent bonds and nonbonding electron pairs of atoms. These are characterized by local minima in the exchange contribution to the electronic gas pressure, *p* ^*X*^(**r**).

Contacts N1∴O2 and H3··O2 in the Z6 conformer, classified as *latent* interactions (as discussed earlier), are accompanied by the formation of regions of reduced negative kinetic pressure *p* ^*S*^(**r**) and negative exchange pressure *p* ^*X*^(**r**) with local minima in the interatomic space (Figure S16). In contrast, for the N3··O3 and H7··O1 contacts, which are also classified as *latent* interactions, these features in the distribution of kinetic and exchange contributions to the internal electronic pressure are less pronounced.

The distribution of the kinetic *p* ^*S*^(**r**) and exchange *p* ^*X*^(**r**) components of electronic pressure in the central molecule of the 7‐molecule cluster is similar to that observed in the individual Z6 zwitterion (Figure S16). The N1···O2 and N3···O2 contacts can be classified as *latent* interactions (as discussed earlier). They are accompanied by the formation of regions of reduced negative kinetic *p* ^*S*^(**r**) pressure and negative exchange *p* ^*X*^(**r**) pressure in the interatomic space (Figure S16) without local minima. The N3···O3 and H7···O1 contacts can also be classified as *latent* (secondary) interactions since such features in the distribution of the kinetic and exchange contributions to the internal electronic pressure are less pronounced.

The distribution of the kinetic *p* ^*S*^(**r**) and exchange *p* ^*X*^(**r**) components of electronic pressure in the experimental ED for the DL‐serine crystal (Figure S17) differs from that of the central molecule in the 7‐molecule cluster and the individual Z6 zwitterion. The main difference in molecule‐in‐crystal lies in the increased values of the kinetic and exchange contributions in the examined interatomic regions and the complete absence of local minima in the exchange contribution.

In the distribution of the electrostatic contribution to electronic pressure, *p*_M_(r), between atoms connected by covalent bonds and completed NCIs (e.g., the intermolecular interaction H3···O1(L) marked in Figure S17), a local minimum of this function is observed along the bond line. In both covalent and completed NCIs, this minimum in electrostatic pressure is shifted from the BCP along the bond line toward the more electronegative atom: from hydrogen to oxygen, from carbon to oxygen, and so on (Figure S17). In these local minima, the electron experiences strong attraction simultaneously to the two atomic nuclei connected by the bond path. The resulting net force (sum of the attractive force toward H and the opposing force toward O) is close to zero (~0.00015 a.u.) but not exactly zero, as the attraction to other nuclei in other directions also contributes. The distribution of *p*_M_(*r*) (like the exchange pressure) does not exhibit extrema corresponding to nonbonding (lone) electron pairs of atoms.

The electronic continuum on maps of total electronic pressure is compressed along the covalent bond lines, forming localized bridges with positive values of *p*(**r**), corresponding to the electronic concentrations of bonding electron pairs. At the same time, the CPs of total pressure, separating these bridges, are shifted from the BCP toward the more electronegative atom (Figure S17). In the interatomic regions, the total pressure assumes negative values. Maxima of *p*(**r**) are visible, corresponding to nonbonding electron pairs of atoms O1, O2, and O3. Their spatial arrangement is consistent with the tetrahedral geometry of atom O3, characteristic of a hydroxyl group, and the planar geometry of atoms O1 and O2, characteristic of a carbonyl group.

In the *p*_M_(*r*) distribution between atoms H3 and O3, which form a six‐membered unclosed pseudocycle, a local minimum of electrostatic pressure is observed precisely along the interatomic line, along with a remote saddle point, distinguishing it from other interactions: H1···O2, H3···O2, and H7···O1, which participate in five‐membered pseudocycles (Figure S17). For the H7···O1 interaction, the formation of a local minimum of electrostatic pressure (~0.00005 a.u.), distant from the interatomic line, is noted. Considering the difference in electronegativity between atoms H3 and O3, the bridge of electrostatic pressure presumably indicates that, despite the absence of an RDG isosurface (Figure 7c) in the interatomic region, H3 and O3 may experience attractive electrostatic interaction. It is noteworthy that the O3/H3 distance is significantly shorter than in the central molecule of the cluster of seven molecules. For this interaction, a typical pattern is observed: along the interatomic H3‐O3 line, the basins of neighboring atoms encroach into the interatomic region, rendering this interaction incomplete [41].

Two possible scenarios can be proposed for the *dynamic* changes in the topology of the electrostatic contribution distribution, *p*_M_(*r*), as atoms H3 and O3 approach each other: (1) An additional local minimum might form within the electrostatic pressure bridge or (2) the existing local minimum along the interatomic line already accounts for the attractive electrostatic interaction between H3 and O3. Confirming one scenario and disproving the other would require comparing the *p* ^M^(**r**) distribution for completed and uncompleted interactions based on theoretical ED in the previously identified serine conformers. However, no software currently exists to perform such an analysis. Additionally, an investigation into the *p* ^M^(**r**) distribution for experimental electron densities of crystals of other molecules, where both completed and uncompleted interactions are observed, could provide clarity. Unfortunately, such a study was beyond the scope of this work.

To explore why an RDG isosurface of 0.6 a.u. does not appear between H3 and O3 in the crystal, single‐point calculations were conducted for the geometries of a seven‐molecule cluster and an individual molecule extracted from the crystal. Analyses of QTAIMC and RDG for the resulting theoretical electron densities revealed the presence of four *latent* interactions: H1···O2, H3···O2, H3···O3, and H7···O1. While the molecule in the crystal exhibited lower values of the kinetic component in intramolecular interatomic regions and a complete absence of local minima in exchange pressure, the distribution of these functions for the theoretical single‐point electron densities of the seven‐molecule cluster and the individual molecule mirrored the patterns observed in the optimized Z6 conformer and the seven‐molecule cluster 7Z6.

The calculation of the difference in ED between the single‐point computed central molecule in a seven‐molecule cluster and an isolated molecule extracted from the crystal enabled visualization of the environmental influence and the redistribution of ED from donor atoms to acceptor atoms during the formation of completed intermolecular interactions. In the single‐point cluster of seven molecules, the H3 and O3 atoms of the central molecule form hydrogen bonding bridges with neighboring molecules. This results in an accumulation of accepted ED on the H3 atom, while the O3 atom redistributes its ED from the interatomic H3‐O3 region toward the bonding path of the intermolecular hydrogen bond, effectively donating its ED to the hydrogen atom of a neighboring molecule (Figure S18). A similar effect on the ED gradient (and consequently RDG) can be expected for the crystal.

It can also be hypothesized that RDG does not detect the interaction between H3···O3 atoms in a crystal due to differences in the distributions of theoretical and experimentally reconstructed electron densities within the framework of the multipole model. In the intramolecular interatomic regions, theoretical ED values are significantly lower than those from the multipole model for the crystal. For instance, the theoretical 0.1 a.u. contour is located roughly at the same position as the 1 a.u. contour in the crystal (Figure S19). This discrepancy implies that for RDG—sign(*λ*_2_)*ρ* diagrams for the crystal, a much wider range of sign(*λ*_2_)*ρ* values needs to be considered compared to the theoretical case. Consequently, the use of the multipole model may lead to the loss of critical information about uncompleted *latent* interactions.

### 4.3 Intermolecular Interactions in a Crystal

**FIGURE 9:**
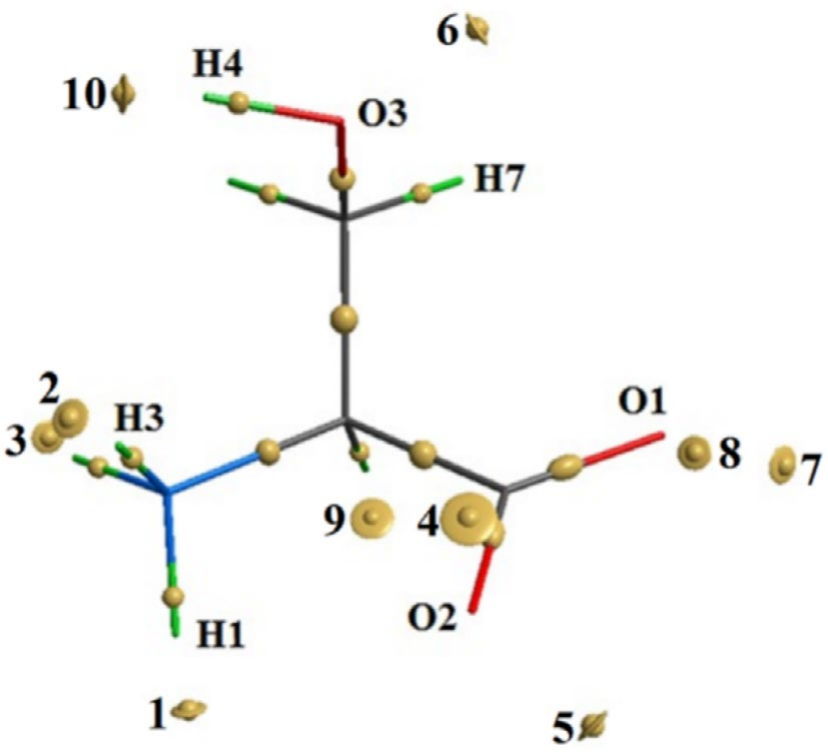
Critical points (CP) of covalent bonds and numbered CP of *completed* intermolecular NCIs. CPs are represented as binding ellipsoids, reflecting the deviation of the ED distribution from cylindrical symmetry (This includes pairs of identical interactions caused by the periodicity of the crystal structure: 1 and 6, 2 and 7, 3 and 8, 4 and 9, 5 and 10.)

QTAIMC analyses (Tables S6 and S7) and RDG of the experimental ED for the crystal of DL‐serine identified 10 intermolecular hydrogen bonds, of which only five are symmetry‐independent (H1···O3 (D), H3···O1 (L), H2···O1 (L), O2···H5 (L), O2···H4 (L)), along with 10 corresponding regions of low ED values and small RDG magnitudes (Figure 9). In contrast, for the seven‐molecule cluster, which lost symmetry elements after optimization, eight completed intermolecular interactions were identified (H1···O3 (D), O3···H1 (D), H3···O1 (L), O1···H3 (L), H2···O1 (L), O1···H2 (L), O2···H4 (L), H4···O2 (L)), along with eight associated regions of low ED values and small RDG magnitudes. In the notation, the second atom belongs to neighboring molecules, with the enantiomer type (D or L) indicated in parentheses.

In the cluster, IQA calculations for all these interactions were not performed due to the high computational resources required, and in a crystal such calculations are now impossible. However, considering the linearity (Tables S6 and S7) and the fact that all these interactions are completed, it is logical to assume that they have relatively low values of intermolecular interaction energy *E*_int_(H···O) compared to the *uncompleted* intramolecular interactions. These *completed* intermolecular interactions in the crystal may hinder vibrations that could bring the pairs of atoms H1···O2, H3···O2, H3···O3, and H7···O1 closer together, leading to the formation of pseudo‐bonding paths in the nonequilibrium structures within the zwitterion. This is because each atom in these four pairs participates in an intermolecular hydrogen bond.

All intermolecular hydrogen bonds in a cluster and crystal are accompanied by regions of reduced values of kinetic pressure *p* ^*S*^(**r**) between the H and O atoms (Figures S20 and S21). However, in the cluster, local minima of exchange pressure *p* ^*X*^(**r**) are observed (Figure S21), whereas in the crystal, saddle points of negative exchange pressure *p* ^*X*^(**r**) coincide with the positions of the CPs of ED (Figure S20). In a crystal, regions of reduced values of total electronic pressure *p*(*r*) and local minima of electrostatic pressure *p* ^M^(*r*) were also identified (Figure S20), located on the interatomic line and shifted toward the more electronegative oxygen atoms.

In intermolecular interatomic spaces, both the kinetic pressure *p* ^*S*^(**r**) and the total pressure *p*(*r*) acquire negative values. Perpendicular to the line of the intermolecular hydrogen bond, these functions increase, while along the direction from the H atom to the O atom, their values locally decrease. For completed intermolecular interactions, no kinetic pressure *p* ^*S*^(**r**) or total pressure *p*(*r*) CPs are formed. A similar pattern was identified for certain linear completed intramolecular interactions in long pseudocycles (8 atoms) in the molecule CH_3_CONH‐Glu‐CONHCH_3_ [85]. However, for shorter pseudocycles (7 atoms), a CP of kinetic pressure *p* ^*S*^(**r**) is formed.

Unlike *completed* and *dynamic* intramolecular interactions, as well as hydrogen bonds in the cluster and even in a single‐point cluster of 7 molecules extracted from the crystal, intermolecular interactions in the crystal are characterized not by a local minimum of exchange pressure *p* ^*X*^(**r**), but by saddle points in the—*p* ^*X*^(**r**) function. This suggests that the type of analyzed ED (theoretical or reconstructed from experimental data using the multipole model) affects the topological details of the exchange pressure *p* ^*X*^(**r**) distribution.

As previously noted, the distribution of kinetic pressure *p* ^*S*^(**r**) and exchange pressure *p* ^*X*^(**r**) in the intermolecular space of the experimental ED for the crystal of DL‐serine differs significantly from that observed for the central molecule in the 7‐molecule cluster and the individual zwitterion Z6. For *latent* interactions H1···O2 and H3···O2 in the crystal, there are no signs of regions with reduced negative kinetic *p* ^*S*^(**r**) or exchange *p* ^*X*^(**r**) pressure values. At the same time, the distribution of kinetic *p* ^*S*^(**r**), exchange *p* ^*X*^(**r**), and total pressure shows strong accumulation of the electronic continuum in the interatomic spaces of intermolecular hydrogen bonds. This is confirmed by the distribution of the ED difference between the central molecule in the single‐point 7‐molecule cluster and the single‐point isolated molecule extracted from the crystal. These observations point to a redistribution of ED from intramolecular interatomic regions toward the regions of intermolecular hydrogen bonds. The absence of an RDG isosurface between the atom pair H3···O3 in the experimental ED, despite its presence in theoretical ED for the central molecule in the cluster and in the zwitterion Z6, can be explained by significantly different ED values in experimental data compared to theory. This discrepancy likely arises from the redistribution of ED into the regions of intermolecular hydrogen bonds or fundamental differences between theoretical and experimental electron densities. In conclusion, the crystal of DL‐serine consistently exhibits three *latent* intramolecular interactions observable via RDG analysis: H1···O2, H3···O2, and H7···O1. Additionally, the interaction H3···O3 is identified through the distribution of electrostatic pressure, although its absence in RDG data is likely caused by either a significant shift of ED toward intermolecular hydrogen bonds or differences between theoretical and experimental electron densities. These four interactions do not influence the structural organization of the serine molecule in the crystal and rarely, if ever, form pseudo‐bond paths during atomic vibrations.

## 5 Conclusions

The application of quantum‐chemical methods based on the ED has demonstrated that, in the neutral serine molecule, its zwitterion, clusters, and molecular crystal, *latent* (incomplete, potential) NCIs can present in addition to covalent hydrogen bonds. Their interaction energies are negative, indicating their attractive (stabilizing) nature.

Quantum‐topological analysis of nonequilibrium structures of conformers of the neutral molecule and zwitterion of serine, arising from atomic displacements during low‐frequency vibrations, allowed *latent* interactions to be classified into two types: *dynamic* and *static*. *Dynamic* interactions influence the structural organization of the molecule by creating a “pulsating” network of atomic contacts, within which pseudo‐bond paths may form or break. Such picture is not observed for *static* interactions.

Analysis of the internal electronic pressure of the electron gas in these systems provided a detailed view of intra‐ and intermolecular interactions. On the kinetic pressure maps, the electronic continuum is compressed along covalent bonds, forming local bridges that correspond to electron pair concentrations in bonding regions. These bridges are separated by saddle points shifted from CPs in the ED toward the more electronegative atom. Lone pairs correspond to maxima of the kinetic pressure. A similar pattern is observed when the molecule transitions to a condensed phase. Covalently bonded atoms are separated in the distribution of the exchange component of the electron continuum pressure, by saddle points, whose positions coincide with the BCPs of the ED. Nonbonding electron pairs are also evident, corresponding to minima in this function. This pattern persists when the molecule transitions to a cluster or crystal.

For completed NCIs, the kinetic component of the electron continuum pressure is negative, and the distribution of the exchange component features local minima, whose positions align with the BCPs of the ED. For *latent* (incomplete) NCIs (both *dynamic* and *static*), the kinetic component of the electron continuum pressure is less negative. The exchange component distribution for *dynamic latent* interactions exhibits local minima, located off the interatomic axis. Such details are absent for *static* interactions.

When the molecule transitions to a condensed phase (a cluster or a crystal), the kinetic component of the electron continuum pressure and the total quantum pressure are positive in covalent bond regions (which are “hard” in respect to external actions) but negative in interatomic regions associated with “soft” NCIs. The exchange component distribution for intramolecular *latent* interactions remains consistent as the molecule transitions to a cluster. However, in the crystal, local minima in the interatomic regions observed in the isolated molecule are absent. Intermolecular hydrogen bonds in the cluster are characterized by the presence of a local minimum in the exchange pressure, whereas in the crystal, they are characterized by saddle points in this function. Thus, the study of these systems demonstrates that the kinetic contribution to the electron continuum pressure exerts the most significant influence on the localization of electron pairs near atoms, while the exchange component reveals the finer details of interatomic interactions.

## Supporting information


Data S1.

## Data Availability

The data that supports the findings of this study are available in the Supporting Information of this article.

## References

[1] J. Černý and P. Hobza , “Non‐Covalent Interactions in Biomacromolecules,” Physical Chemistry Chemical Physics 9, no. 39 (2007): 5291–5303. 17914464 10.1039/b704781a

[2] E. Frieden , “Non‐Covalent Interactions: Key to Biological Flexibility and Specificity,” Journal of Chemical Education 52, no. 12 (1975): 754. 172524 10.1021/ed052p754

[3] G. D. Rose , P. J. Fleming , J. R. Banavar , and A. Maritan , “A Backbone‐Based Theory of Protein Folding,” Proceedings of the National Academy of Sciences 103, no. 45 (2006): 16623–16633. 10.1073/pnas.0606843103 PMC1636505 17075053

[4] C. B. Anfinsen , “Principles That Govern the Folding of Protein Chains,” Science 181, no. 4096 (1973): 223–230. 4124164 10.1126/science.181.4096.223

[5] C. N. Pace , B. A. Shirley , M. McNutt , and K. Gajiwala , “Forces Contributing to the Conformational Stability of Proteins,” FASEB Journal 10, no. 1 (1996): 75–83. 8566551 10.1096/fasebj.10.1.8566551

[6] S. Deechongkit , H. Nguyen , E. T. Powers , P. E. Dawson , M. Gruebele , and J. W. Kelly , “Context‐Dependent Contributions of Backbone Hydrogen Bonding to β‐Sheet Folding Energetics,” Nature 430, no. 6995 (2004): 101–105. 15229605 10.1038/nature02611

[7] E. Fischer , “Influence of Configuration on the Action of Enzymes,” Berichte der Deutschen Chemischen Gesellschaft 27 (1894): 2985–2993.

[8] B. Nidetzky , C. EIS , and M. Albert , “Role of Non‐Covalent Enzyme–Substrate Interactions in the Reaction Catalysed by Cellobiose Phosphorylase From *Cellulomonas uda* ,” Biochemical Journal 351, no. 3 (2000): 649–659. 11042119 PMC1221404

[9] K. Iijima , K. Tanaka , and S. Onuma , “Main Conformer of Gaseous Glycine: Molecular Structure and Rotational Barrier From Electron Diffraction Data and Rotational Constants,” Journal of Molecular Structure 246, no. 3–4 (1991): 257–266.

[10] R. D. Brown , P. D. Godfrey , J. W. Storey , and M.‐P. Bassez , “Microwave Spectrum and Conformation of Glycine,” Journal of the Chemical Society, Chemical Communications 13 (1978): 547–548.

[11] R. D. Suenram and F. Lovas , “Millimeter Wave Spectrum of Glycine,” Journal of Molecular Spectroscopy 72, no. 3 (1978): 372–382.

[12] R. D. Suenram and F. J. Lovas , “Millimeter Wave Spectrum of Glycine. A New Conformer,” Journal of the American Chemical Society 102, no. 24 (1980): 7180–7184.

[13] A. G. Csaszar , “Conformers of Gaseous Glycine,” Journal of the American Chemical Society 114, no. 24 (1992): 9568–9575.

[14] P. D. Godfrey and R. D. Brown , “Shape of Glycine,” Journal of the American Chemical Society 117, no. 7 (1995): 2019–2023.

[15] Y.‐J. Kuan , S. B. Charnley , H.‐C. Huang , W.‐L. Tseng , and Z. Kisiel , “Interstellar Glycine,” Astrophysical Journal 593, no. 2 (2003): 848–867.

[16] K. Iijima and B. Beagley , “An Electron Diffraction Study of Gaseous α‐Alanine, NH_2_CHCH_3_CO_2_H,” Journal of Molecular Structure 248, no. 1–2 (1991): 133–142.

[17] P. Godfrey , S. Firth , L. Hatherley , R. Brown , and A. Pierlot , “Millimeter‐Wave Spectroscopy of Biomolecules: Alanine,” Journal of the American Chemical Society 115, no. 21 (1993): 9687–9691.

[18] S. Stepanian , I. Reva , E. Radchenko , and L. Adamowicz , “Conformational Behavior of α‐Alanine. Matrix‐Isolation Infrared and Theoretical DFT and Ab Initio Study,” Journal of Physical Chemistry A 102, no. 24 (1998): 4623–4629.

[19] S. Stepanian , I. Reva , E. Radchenko , and L. Adamowicz , “Combined Matrix‐Isolation Infrared and Theoretical DFT and Ab Initio Study of the Nonionized Valine Conformers,” Journal of Physical Chemistry A 103, no. 22 (1999): 4404–4412.

[20] I. Reva , S. Stepanian , A. Plokhotnichenko , E. Radchenko , G. Sheina , and Y. P. Blagoi , “Infrared Matrix Isolation Studies of Amino Acids. Molecular Structure of Proline,” Journal of Molecular Structure 318 (1994): 1–13.

[21] I. D. Reva , A. M. Plokhotnichenko , S. G. Stepanian , et al., “The Rotamerization of Conformers of Glycine Isolated in Inert Gas Matrices. An Infrared Spectroscopic Study,” Chemical Physics Letters 232, no. 1–2 (1995): 141–148.

[22] L. Hedstrom , “Serine Protease Mechanism and Specificity,” Chemical Reviews 102, no. 12 (2002): 4501–4524. 12475199 10.1021/cr000033x

[23] K. He and W. D. Allen , “Conformers of Gaseous Serine,” Journal of Chemical Theory and Computation 12, no. 8 (2016): 3571–3582. 27294314 10.1021/acs.jctc.6b00314

[24] R. Miao , C. Jin , G. Yang , J. Hong , C. Zhao , and L. Zhu , “Comprehensive Density Functional Theory Study on Serine and Related Ions in Gas Phase: Conformations, Gas Phase Basicities, and Acidities,” Journal of Physical Chemistry A 109, no. 10 (2005): 2340–2349. 16839004 10.1021/jp0453919

[25] A. E. Counterman and D. E. Clemmer , “Magic Number Clusters of Serine in the Gas Phase,” Journal of Physical Chemistry B 105, no. 34 (2001): 8092–8096.

[26] S. Stepanian , I. Reva , E. Radchenko , et al., “Matrix‐Isolation Infrared and Theoretical Studies of the Glycine Conformers,” Journal of Physical Chemistry A 102, no. 6 (1998): 1041–1054.

[27] M. S. Lehmann , T. F. Koetzle , and W. C. Hamilton , “Precision Neutron Diffraction Structure Determination of Protein and Nucleic Acid Components. I. Crystal and Molecular Structure of the Amino Acid L‐Alanine,” Journal of the American Chemical Society 94, no. 8 (1972): 2657–2660. 5017418 10.1021/ja00763a016

[28] J. Ireta , J. Neugebauer , and M. Scheffler , “On the Accuracy of DFT for Describing Hydrogen Bonds: Dependence on the Bond Directionality,” Journal of Physical Chemistry A 108, no. 26 (2004): 5692–5698.

[29] P. A. Wood , F. H. Allen , and E. Pidcock , “Hydrogen‐Bond Directionality at the Donor H Atom—Analysis of Interaction Energies and Database Statistics,” CrystEngComm 11, no. 8 (2009): 1563–1571.

[30] S. S. Batsanov , “Van Der Waals Radii of Elements,” Inorganic Materials 37, no. 9 (2001): 871–885.

[31] L. Jiang and L. Lai , “CH··· O Hydrogen Bonds at Protein‐Protein Interfaces* 210,” Journal of Biological Chemistry 277, no. 40 (2002): 37732–37740. 12119293 10.1074/jbc.M204514200

[32] G. R. Desiraju , “The C− H··· O Hydrogen Bond: Structural Implications and Supramolecular Design,” Accounts of Chemical Research 29, no. 9 (1996): 441–449. 23618410 10.1021/ar950135n

[33] G. R. Desiraju and T. Steiner , The Weak Hydrogen Bond: In Structural Chemistry and Biology (International Union of Crystal, 2001).

[34] R. F. Bader , “Atoms in Molecules,” Accounts of Chemical Research 18, no. 1 (1985): 9–15.

[35] G. Runtz , R. Bader , and R. Messer , “Definition of Bond Paths and Bond Directions in Terms of the Molecular Charge Distribution,” Canadian Journal of Chemistry 55, no. 16 (1977): 3040–3045.

[36] R. F. Bader , “Bond Paths Are Not Chemical Bonds,” Journal of Physical Chemistry A 113, no. 38 (2009): 10391–10396. 19722600 10.1021/jp906341r

[37] R. F. Bader , “A Bond Path: A Universal Indicator of Bonded Interactions,” Journal of Physical Chemistry A 102, no. 37 (1998): 7314–7323.

[38] E. R. Johnson , S. Keinan , P. Mori‐Sánchez , J. Contreras‐García , A. J. Cohen , and W. Yang , “Revealing Noncovalent Interactions,” Journal of the American Chemical Society 132, no. 18 (2010): 6498–6506. 20394428 10.1021/ja100936w PMC2864795

[39] J. Contreras‐García , E. R. Johnson , S. Keinan , et al., “NCIPLOT: A Program for Plotting Noncovalent Interaction Regions,” Journal of Chemical Theory and Computation 7, no. 3 (2011): 625–632. 21516178 10.1021/ct100641a PMC3080048

[40] A. Otero‐De‐La‐Roza , E. R. Johnson , and J. Contreras‐García , “Revealing Non‐Covalent Interactions in Solids: NCI Plots Revisited,” Physical Chemistry Chemical Physics 14, no. 35 (2012): 12165–12172. 22850808 10.1039/c2cp41395g

[41] E. V. Bartashevich , Á. M. Pendás , and V. G. Tsirelson , “An Anatomy of Intramolecular Atomic Interactions in Halogen‐Substituted Trinitromethanes,” Physical Chemistry Chemical Physics 16, no. 31 (2014): 16780–16789. 25001471 10.1039/c4cp01257g

[42] E. Suarez , N. Diaz , and D. Suarez , “Entropy Calculations of Single Molecules by Combining the Rigid–Rotor and Harmonic‐Oscillator Approximations With Conformational Entropy Estimations From Molecular Dynamics Simulations,” Journal of Chemical Theory and Computation 7, no. 8 (2011): 2638–2653. 26606637 10.1021/ct200216n

[43] K. Moritsugu , O. Miyashita , and A. Kidera , “Vibrational Energy Transfer in a Protein Molecule,” Physical Review Letters 85, no. 18 (2000): 3970–3973. 11041973 10.1103/PhysRevLett.85.3970

[44] M. Freindorf , E. Kraka , and D. Cremer , “A Comprehensive Analysis of Hydrogen Bond Interactions Based on Local Vibrational Modes,” International Journal of Quantum Chemistry 112, no. 19 (2012): 3174–3187.

[45] J. González , R. Martínez , J. A. Fernández , and J. Millan , “Conformational Landscape of Isolated Capped Amino Acids: On the Nature of Non‐Covalent Interactions,” European Physical Journal D 71 (2017): 1–12.

[46] A. A. Howard , G. S. Tschumper , and N. I. Hammer , “Effects of Hydrogen Bonding on Vibrational Normal Modes of Pyrimidine,” Journal of Physical Chemistry A 114, no. 25 (2010): 6803–6810. 20527867 10.1021/jp101267w

[47] A. M. Pendás , M. Blanco , and E. Francisco , “Two‐Electron Integrations in the Quantum Theory of Atoms in Molecules,” Journal of Chemical Physics 120, no. 10 (2004): 4581–4592. 15267317 10.1063/1.1645788

[48] M. Blanco , A. Martín Pendás , and E. Francisco , “Interacting Quantum Atoms: A Correlated Energy Decomposition Scheme Based on the Quantum Theory of Atoms in Molecules,” Journal of Chemical Theory and Computation 1, no. 6 (2005): 1096–1109. 26631653 10.1021/ct0501093

[49] E. Francisco , A. Martín Pendás , and M. Blanco , “A Molecular Energy Decomposition Scheme for Atoms in Molecules,” Journal of Chemical Theory and Computation 2, no. 1 (2006): 90–102. 26626383 10.1021/ct0502209

[50] A. Martín Pendás , E. Francisco , and M. Blanco , “Binding Energies of First Row Diatomics in the Light of the Interacting Quantum Atoms Approach,” Journal of Physical Chemistry A 110, no. 47 (2006): 12864–12869. 17125302 10.1021/jp063607w

[51] A. M. Pendás , M. A. Blanco , and E. Francisco , “Chemical Fragments in Real Space: Definitions, Properties, and Energetic Decompositions,” Journal of Computational Chemistry 28, no. 1 (2007): 161–184. 17061243 10.1002/jcc.20469

[52] V. Tognetti and L. Joubert , “On the Physical Role of Exchange in the Formation of an Intramolecular Bond Path Between Two Electronegative Atoms,” Journal of Chemical Physics 138, no. 2 (2013): 024102. 23320663 10.1063/1.4770495

[53] J. Tao , G. Vignale , and I. Tokatly , “Quantum Stress Focusing in Descriptive Chemistry,” Physical Review Letters 100, no. 20 (2008): 206405. 18518562 10.1103/PhysRevLett.100.206405

[54] R. G. Parr and W. Yang , “Density‐Functional Theory of the Electronic Structure of Molecules,” Annual Review of Physical Chemistry 46, no. 1 (1995): 701–728. 10.1146/annurev.pc.46.100195.003413 24341393

[55] I. E. Tamm , Fundamentals of Electricity Theory (Mir Publishers, 1929).

[56] V. G. Tsirelson , A. I. Stash , and I. V. Tokatly , “Bonding in Molecular Crystals From the Local Electronic Pressure Viewpoint,” Molecular Physics 114, no. 7–8 (2016): 1260–1269.

[57] A. A. Astakhov , A. I. Stash , and V. G. Tsirelson , “Improving Approximate Determination of the Noninteracting Electronic Kinetic Energy Density From Electron Density,” International Journal of Quantum Chemistry 116, no. 3 (2016): 237–246.

[58] D. A. Kirzhnits , “Quantum Corrections to the Thomas‐Fermi Equation,” Journal of Experimental and Theoretical Physics 32, no. 1 (1957): 115–123.

[59] A. D. Becke , “Density‐Functional Exchange‐Energy Approximation With Correct Asymptotic Behavior,” Physical Review A 38, no. 6 (1988): 3098–3100. 10.1103/physreva.38.3098 9900728

[60] A. D. Becke , “A New Mixing of Hartree–Fock and Local Density‐Functional Theories,” Journal of Chemical Physics 98, no. 2 (1993): 1372–1377.

[61] W. Yang , R. G. Parr , and C. Lee , “Various Functionals for the Kinetic Energy Density of an Atom or Molecule,” Physical Review A 34, no. 6 (1986): 4586–4590. 10.1103/physreva.34.4586 9897838

[62] R. Improta , V. Barone , K. N. Kudin , and G. E. Scuseria , “The Conformational Behavior of Polyglycine as Predicted by a Density Functional Model With Periodic Boundary Conditions,” Journal of Chemical Physics 114, no. 6 (2001): 2541–2549.

[63] A. V. Marenich , C. J. Cramer , and D. G. Truhlar , “Universal Solvation Model Based on Solute Electron Density and on a Continuum Model of the Solvent Defined by the Bulk Dielectric Constant and Atomic Surface Tensions,” Journal of Physical Chemistry B 113, no. 18 (2009): 6378–6396. 19366259 10.1021/jp810292n

[64] A. V. Marenich , C. J. Cramer , and D. G. Truhlar , “Performance of SM6, SM8, and SMD on the SAMPL1 Test Set for the Prediction of Small‐Molecule Solvation Free Energies,” Journal of Physical Chemistry B 113, no. 14 (2009): 4538–4543. 19253989 10.1021/jp809094y

[65] B. Dittrich , C. B. Hübschle , M. Messerschmidt , R. Kalinowski , D. Girnt , and P. Luger , “The Invariom Model and Its Application: Refinement of D, L‐Serine at Different Temperatures and Resolution,” Acta Crystallographica Section A: Foundations of Crystallography 61, no. 3 (2005): 314–320. 15846034 10.1107/S0108767305005039

[66] S. Spicher and S. Grimme , “Single‐Point Hessian Calculations for Improved Vibrational Frequencies and Rigid‐Rotor‐Harmonic‐Oscillator Thermodynamics,” Journal of Chemical Theory and Computation 17, no. 3 (2021): 1701–1714. 33554604 10.1021/acs.jctc.0c01306

[67] F. Biegler‐König and J. Schönbohm , “Update of the AIM2000‐Program for Atoms in Molecules,” Journal of Computational Chemistry 23, no. 15 (2002): 1489–1494. 12370951 10.1002/jcc.10085

[68] T. Lu and F. Chen , “Multiwfn: A Multifunctional Wavefunction Analyzer,” Journal of Computational Chemistry 33, no. 5 (2012): 580–592. 22162017 10.1002/jcc.22885

[69] T. Keith , “AIMAll, Revision 17.01. 25,” 2017, TK Gristmill Software.

[70] A. I. Stash and V. G. Tsirelson , “Developing WinXPRO: A Software for Determination of the Multipole‐Model‐Based Properties of Crystals,” Journal of Applied Crystallography 47, no. 6 (2014): 2086–2089.

[71] V. Korotenko , P. Langrzyk , and H. Zipse , “Computational Prediction of One‐Electron Oxidation Potentials for Cytosine and Uracil Epigenetic Derivatives,” Journal of Physical Chemistry A 129 (2025): 4339–4356. 40199460 10.1021/acs.jpca.4c06944 PMC12105625

[72] V. Korotenko and H. Zipse , “The Stability of Oxygen‐Centered Radicals and Its Response to Hydrogen Bonding Interactions,” Journal of Computational Chemistry 45, no. 2 (2024): 101–114. 37747356 10.1002/jcc.27221

[73] S. Gronert and R. A. O'Hair , “Ab Initio Studies of Amino Acid Conformations. 1. The Conformers of Alanine, Serine, and Cysteine,” Journal of the American Chemical Society 117, no. 7 (1995): 2071–2081.

[74] I. S. Jeon , D. S. Ahn , S. W. Park , S. Lee , and B. Kim , “Structures and Isomerization of Neutral and Zwitterion Serine–Water Clusters: Computational Study,” International Journal of Quantum Chemistry 101, no. 1 (2005): 55–66.

[75] S. Jarmelo and R. Fausto , “Entropy Effects in Conformational Distribution and Conformationally Dependent UV‐Induced Photolysis of Serine Monomer Isolated in Solid Argon,” Journal of Molecular Structure 786, no. 2–3 (2006): 175–181.

[76] B. Lambie , R. Ramaekers , and G. Maes , “Conformational Behavior of Serine: An Experimental Matrix‐Isolation FT‐IR and Theoretical DFT (B3LYP)/6‐31++ G** Study,” Journal of Physical Chemistry A 108, no. 47 (2004): 10426–10433.

[77] Y. Zubavichus , O. Fuchs , L. Weinhardt , et al., “Soft X‐Ray‐Induced Decomposition of Amino Acids: An XPS, Mass Spectrometry, and NEXAFS Study,” Radiation Research 161, no. 3 (2004): 346–358. 15108703 10.1667/rr3114.1

[78] P. Ehrenfreund , M. Bernstein , J. Dworkin , S. Sandford , and L. Allamandola , “The Photostability of Amino Acids in Space,” Astrophysical Journal 550, no. 1 (2001): L95.

[79] B. Lambie , R. Ramaekers , and G. Maes , “On the Contribution of Intramolecular H‐Bonding Entropy to the Conformational Stability of Alanine Conformations,” Spectrochimica Acta Part A: Molecular and Biomolecular Spectroscopy 59, no. 6 (2003): 1387–1397. 12659907 10.1016/s1386-1425(02)00353-0

[80] A. Perczel and I. G. Csizmadia , “Searching for the Simplest Structural Units to Describe the Three‐Dimensional Structure of Proteins,” International Reviews in Physical Chemistry 14, no. 1 (1995): 127–168.

[81] J. Tomasi , B. Mennucci , and E. Cancès , “The IEF Version of the PCM Solvation Method: An Overview of a New Method Addressed to Study Molecular Solutes at the QM Ab Initio Level,” Journal of Molecular Structure: THEOCHEM 464, no. 1–3 (1999): 211–226.

[82] M. M. Quesada‐Moreno , J. R. Avilés‐Moreno , A. Á. Márquez‐García , F. Partal‐Ureña , and J. J. L. González , “L‐Serine in Aqueous Solutions at Different pH: Conformational Preferences and Vibrational Spectra of Cationic, Anionic and Zwitterionic Species,” Journal of Molecular Structure 1046 (2013): 136–146.

[83] P. Zhu , G. Yang , M. R. Poopari , Z. Bie , and Y. Xu , “Conformations of Serine in Aqueous Solutions as Revealed by Vibrational Circular Dichroism,” ChemPhysChem 13, no. 5 (2012): 1272–1281. 22334359 10.1002/cphc.201101003

[84] S. Ebrahimi , H. A. Dabbagh , and K. Eskandari , “Nature of Intramolecular Interactions of Vitamin C in View of Interacting Quantum Atoms: The Role of Hydrogen Bond Cooperativity on Geometry,” Physical Chemistry Chemical Physics 18, no. 27 (2016): 18278–18288. 27332782 10.1039/c6cp01678b

[85] A. R. Kazimir , A. N. Egorova , and V. G. Tsirelson , “Pressure and Forces in the Electronic Subsystem of C7 Conformers of Glutamic Acid Diamide in the Region of Non‐Covalent Interactions,” Advances in Chemistry and Chemical Technology 33, no. 2 (2019): 20–22.

