# Supplementary material for: Unveiling Hidden Intramolecular Non‐Covalent Interactions in a Neutral Serine, Its Zwitterion, Cluster, and Crystal by Features of Electron Density
Source: J Comput Chem. 2025 Jun 30;46(17):e70134. doi: 10.1002/jcc.70134 (PMC12207953; doi:10.1002/jcc.70134)
Supplement: Supplementary file 1 — Data S1. [file JCC-46-0-s001.pdf]

Supporting Information

«Unveiling Hidden Intramolecular Non-Covalent Interactions in a Neutral Serine, its Zwitterion, Cluster, and Crystal by Features of Electron Density»

Vasilii Korotenko, [1,2*] Anna Egorova, [3] Vladimir Tsirelson [3]

[1] Thermal Separation Processes, Hamburg University of Technology (TUHH), Denickestraße 22, 21073 Hamburg, Germany

[2] Department of Chemistry, LMU München, Butenandtstrasse 5-13, 81377 München, Germany

[3] Quantum Chemistry Department, D. I. Mendeleev University, Miusskaya Sq. 9, 125047 Moscow, Russia

* Correspondence to: Vasilii Korotenko, E-mail: vasilii.korotenko@tuhh.de

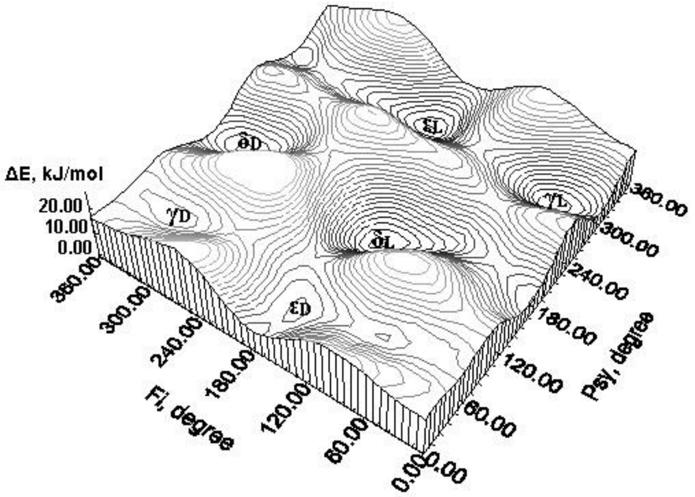

Figure S 1. Potential energy surface of the main chain in serine molecule (method B3LYP/6-31G**.)

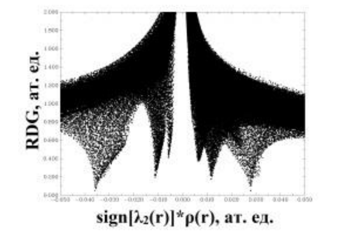	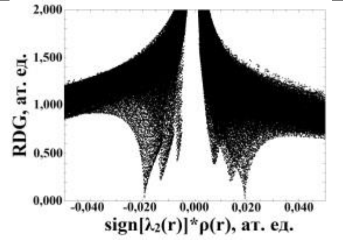	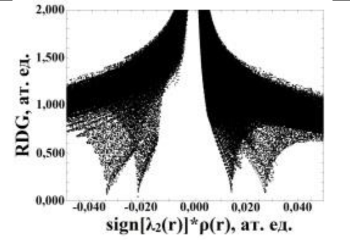
N1, 0.0	N2, 0.1	N3, 1.6
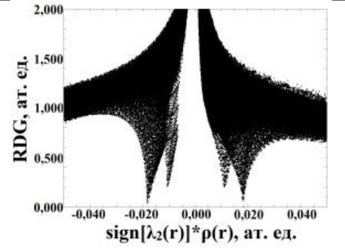	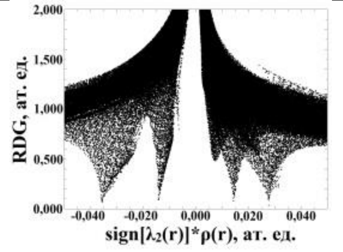	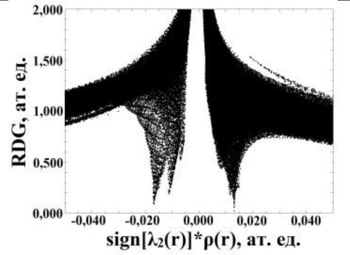
N4, 3.7	N5, ΔE =6.3	N6, 6.5
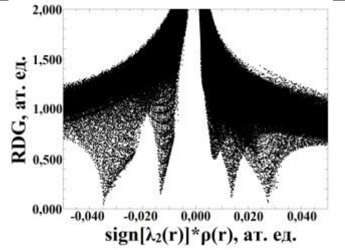	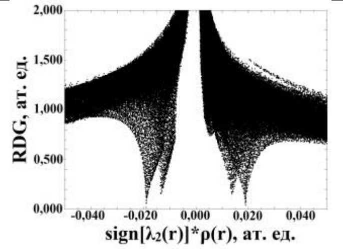	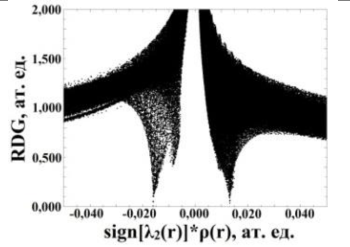
N7, 6.6	N8, 6.7	N9, 9.3

Figure S 2. Diagram: abscissa axis sign[λ_2(r)]*ρ(r) a.u., ordinate axis RDG a.u.

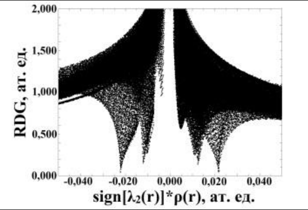	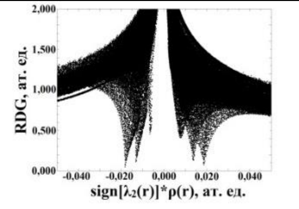	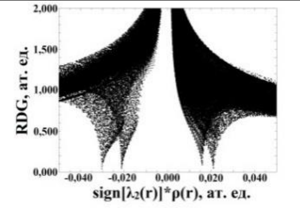	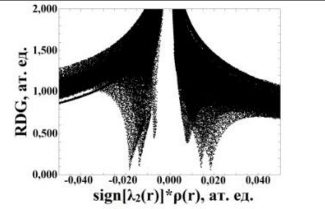
Z1, 0.0	Z2, 0.4	Z3, 2.5	Z4, 2.9
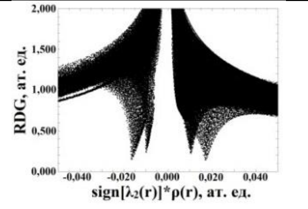	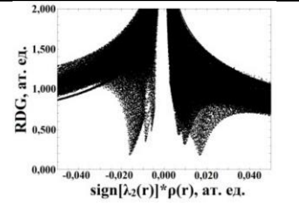	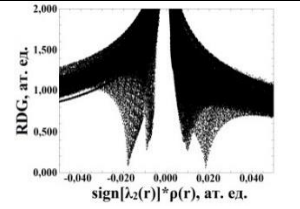	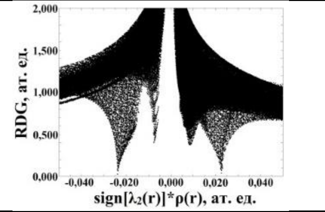
Z5, 3.7	Z6, 4.4	Z7, 4.5	Z8, 5.4

Figure S 3. Diagram: the x-axis represents sign[λ_2(r)] *ρ(r) (a.u.), and the y-axis represents RDG (a.u.).

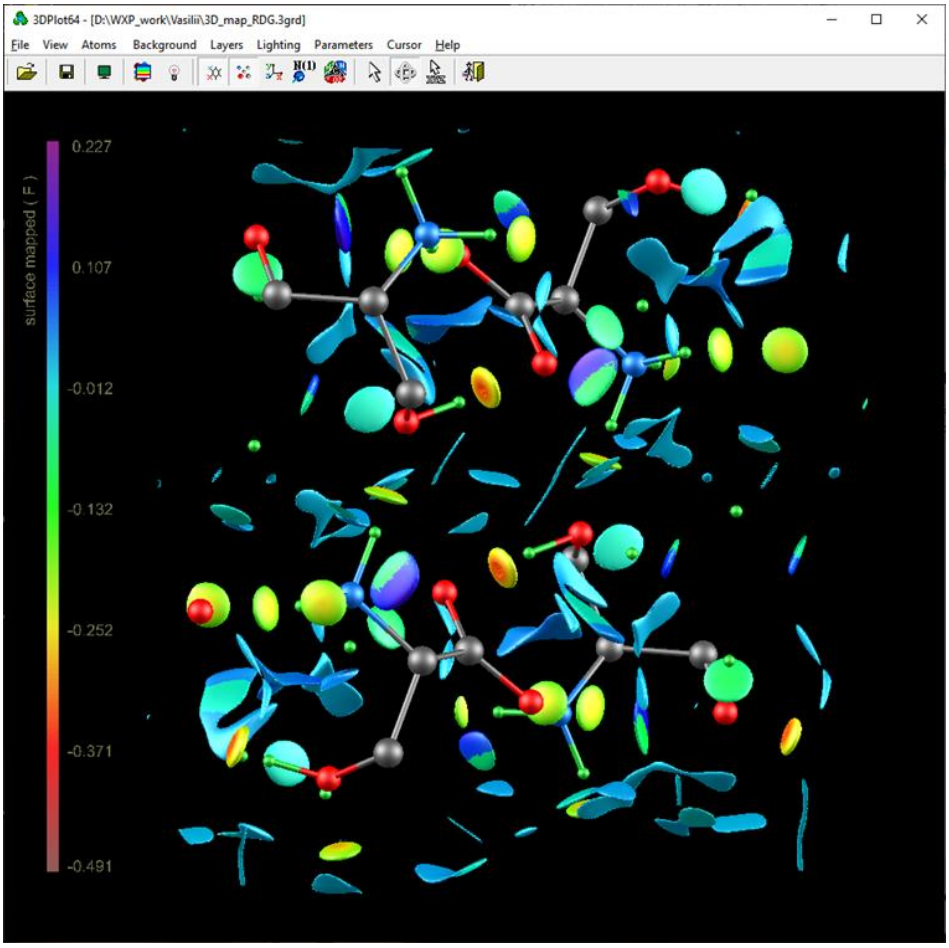

Figure S 4. RDG analysis using WinXPro program for the DL-crystal at the B3LYP/POB-TZVP. (Picture by Adam Stash)

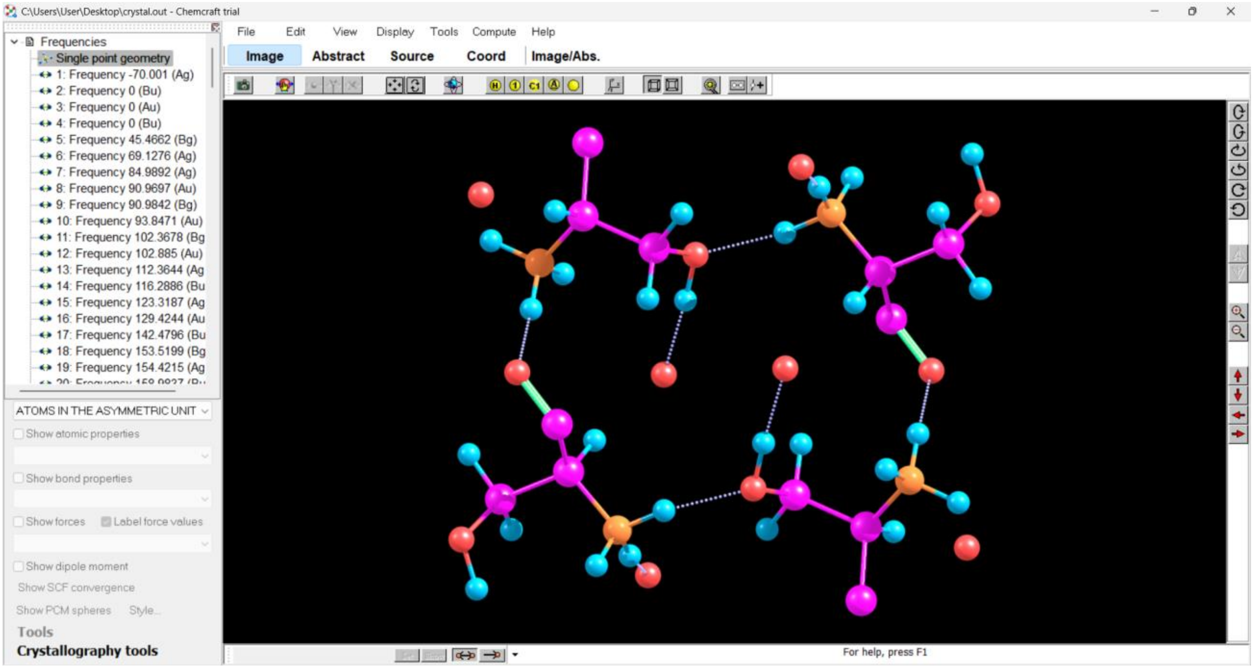

Figure S 5. Frequencies analysis for the optimized crystal at the B3LYP/POB-TZVP level.

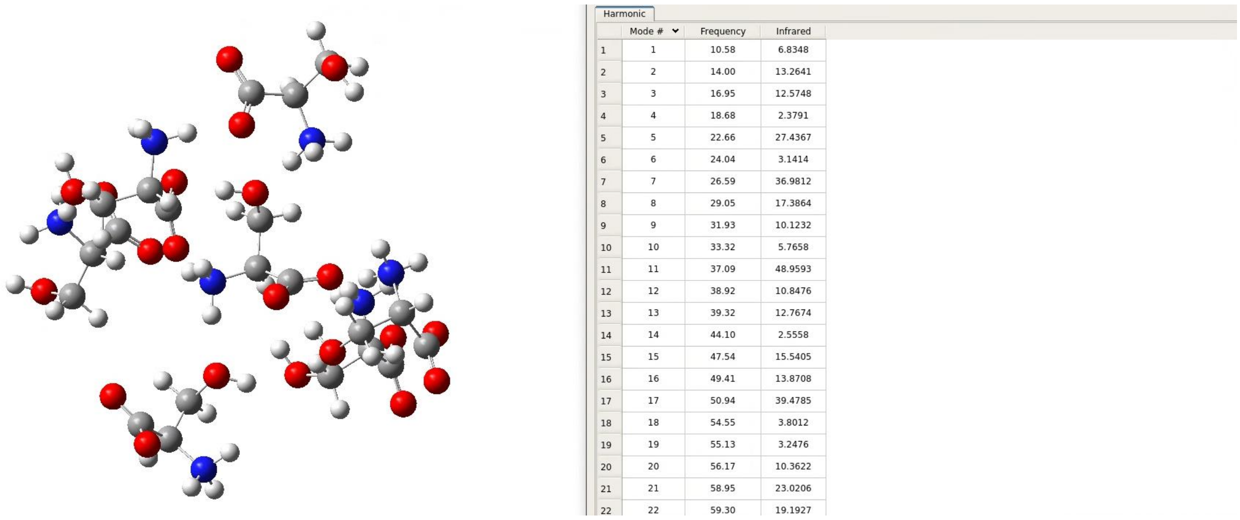

	Mode #	Frequency	Infrared
1	1	10.58	6.8348
2	2	14.00	13.2641
3	3	16.95	12.5748
4	4	18.68	2.3791
5	5	22.66	27.4367
6	6	24.04	3.1414
7	7	26.59	36.9812
8	8	29.05	17.3864
9	9	31.93	10.1232
10	10	33.32	5.7658
11	11	37.09	48.9593
12	12	38.92	10.8476
13	13	39.32	12.7674
14	14	44.10	2.5558
15	15	47.54	15.5405
16	16	49.41	13.8708
17	17	50.94	39.4785
18	18	54.55	3.8012
19	19	55.13	3.2476
20	20	56.17	10.3622
21	21	58.95	23.0206
22	22	59.30	19.1927

Figure S 6. Frequencies analysis for the optimized 7Z6 cluster at the B3LYP/6-311++G(d,p) level.

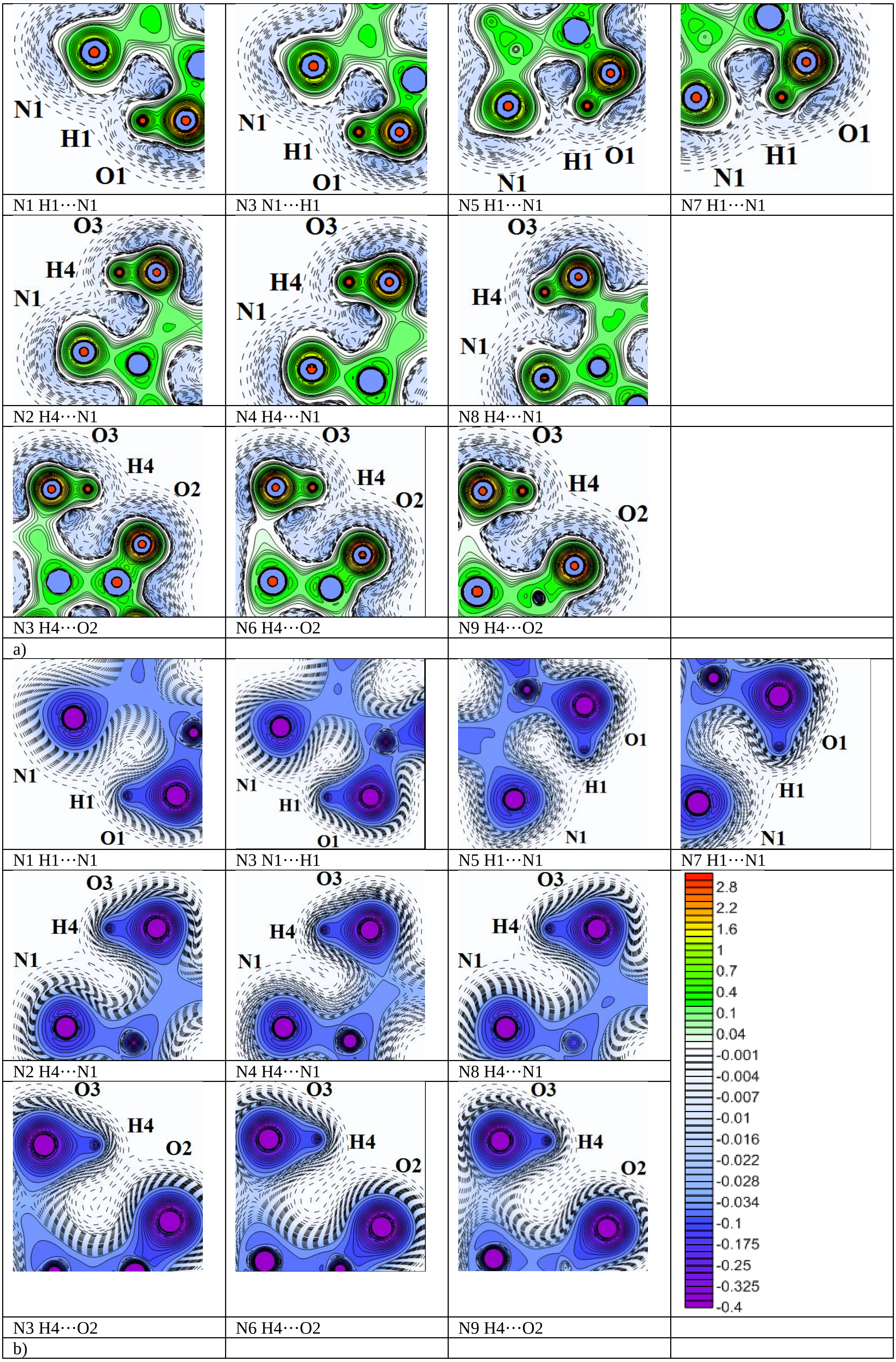

Figure S 7. Distribution of kinetic (a) and exchange (b) parts of the internal electron pressure in conformers N1-N9 of a neutral serine molecule: **completed** NCIs. The cross-sectional planes are drawn through the indicated atoms. Dotted lines correspond to values from -0.034 to -0.010 a.u. in 0.002 a.u. steps; -0.010 to 0 a.u. in 0.001 a.u. steps.; solid lines are -0.400 to -0.050 a.u. in steps of 0.025 a.u.; 0 to 0.1 in steps of 0.002 a.u.; 0.1 to 1.0 in steps of 0.1; 1.0 a.u. to 3.0 a.u. in steps of 0.2 a.u.

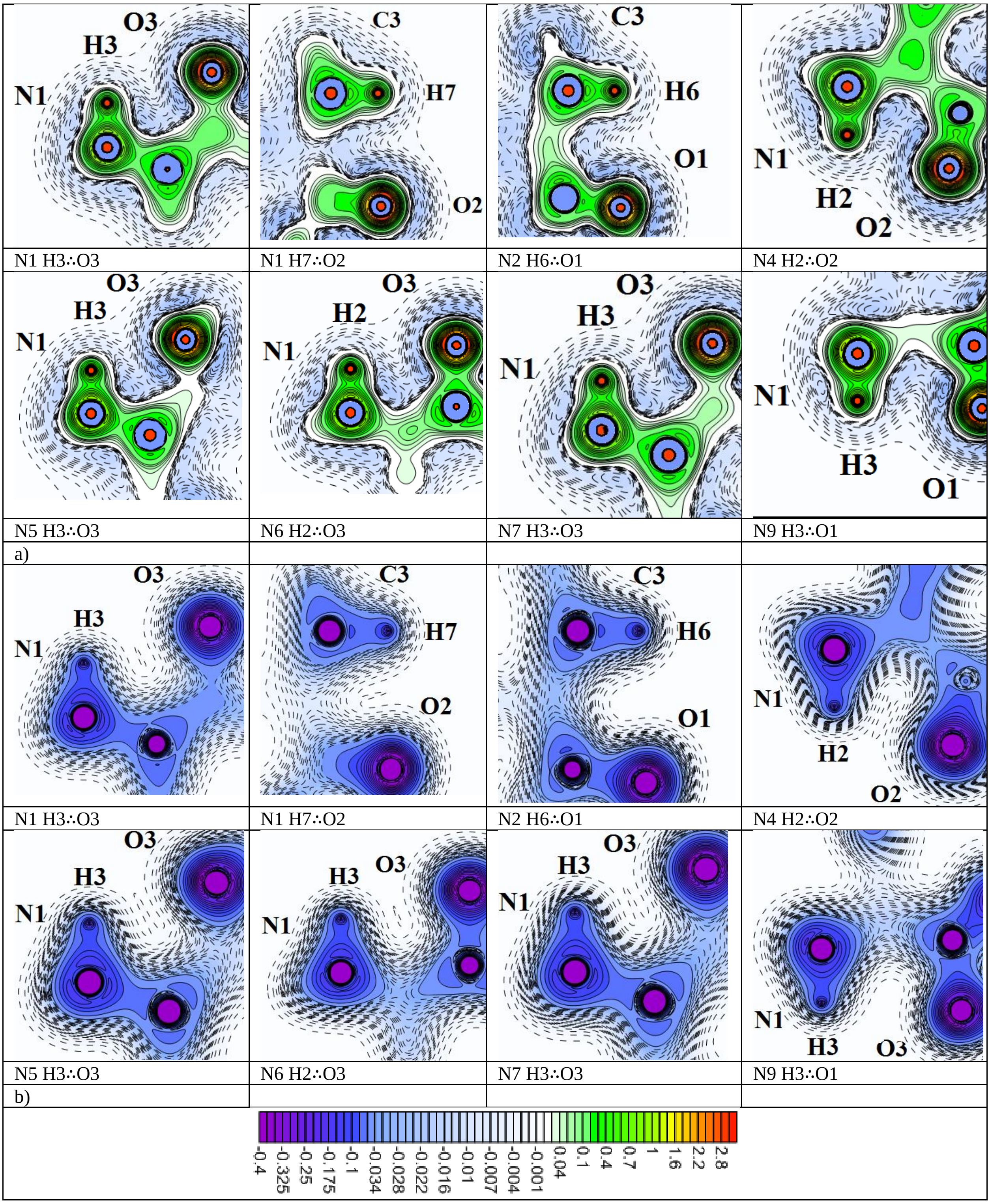

Figure S 8. Distribution of the kinetic (a) and exchange (b) components of the internal electronic pressure in the conformers N1-N9 of the neutral serine molecule: **dynamic** latent NCIs. Cross-sectional planes are drawn through the specified atoms. Dashed lines correspond to values from -0.034 to -0.010 atomic units (a.u.) with a step of 0.002 a.u.; from -0.010 to 0 a.u. with a step of 0.001 a.u. Solid lines represent values from -0.400 to -0.050 a.u. with a step of 0.025 a.u.; from 0 to 0.1 a.u. with a step of 0.002 a.u.; from 0.1 to 1.0 a.u. with a step of 0.1 a.u.; and from 1.0 to 3.0 a.u. with a step of 0.2 a.u.

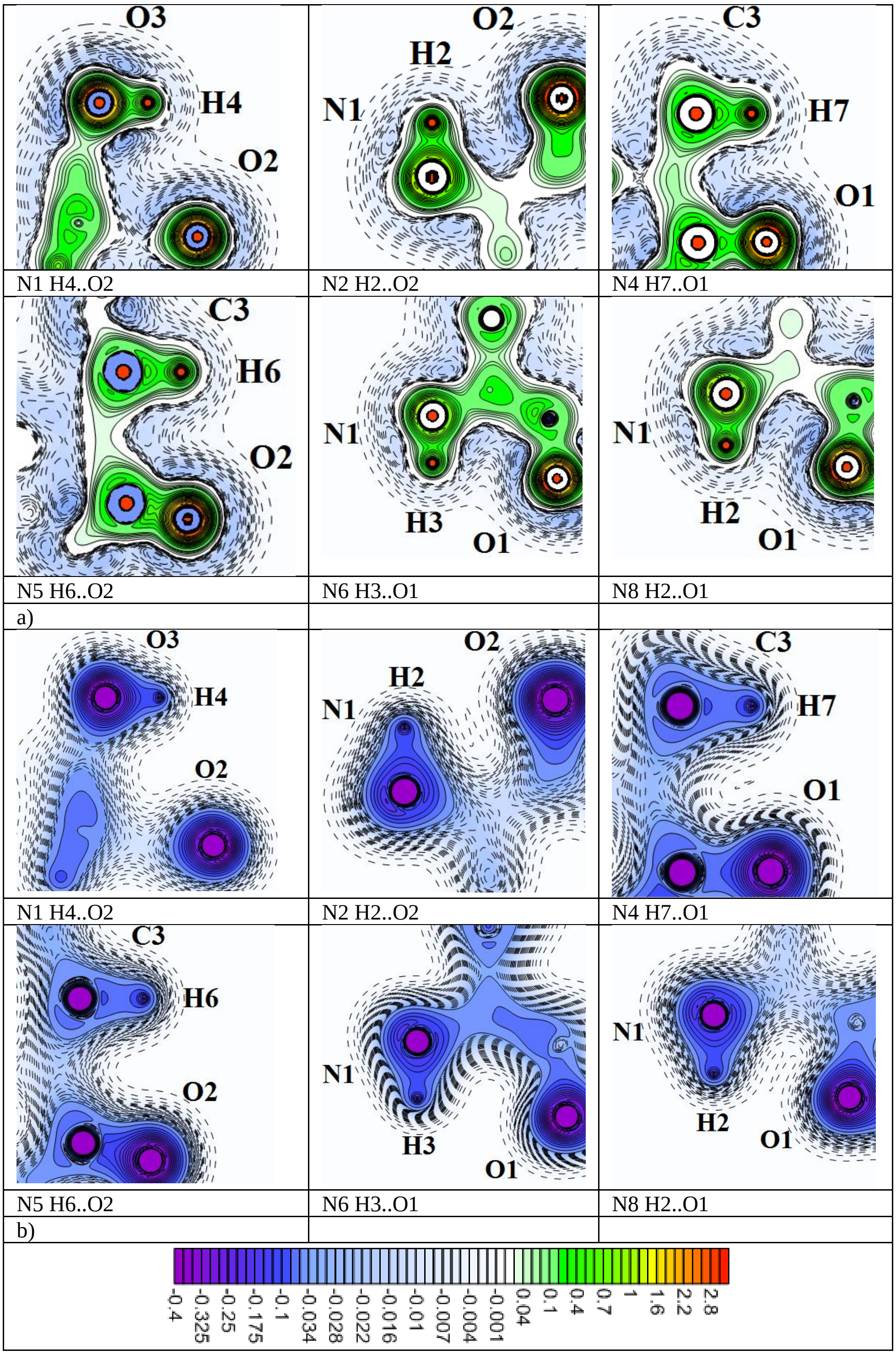

Figure S 9. Distribution of the kinetic (a) and exchange (b) components of the internal electronic pressure in the conformers N1-N9 of the neutral serine molecule: **static latent** NCIs. Cross-sectional planes are drawn through the specified atoms. Dashed lines correspond to values from -0.034 to -0.010 a.u. with a step of 0.002 a.u.; from -0.010 to 0 a.u. with a step of 0.001 a.u. Solid lines represent values from -0.400 to -0.050 a.u. with a step of 0.025 a.u.; from 0 to 0.1 a.u. with a step of 0.002 a.u.; from 0.1 to 1.0 a.u. with a step of 0.1 a.u.; and from 1.0 to 3.0 a.u. with a step of 0.2 a.u.

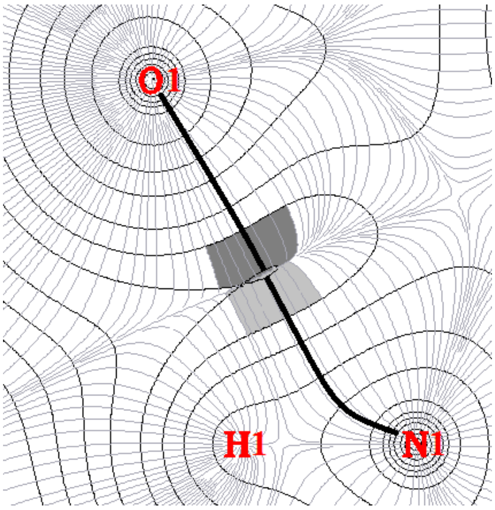

Figure S 10. ED gradient lines and its isolines for the N1-H1...O1 fragment in the nonequilibrium conformer structure at 60 cm^{-1}, when displaced against the vector direction. The binding pathway is depicted by the thick black line. In the region of atom pools contacting: a part of the ED basin of the O1 atom is marked in dark gray, a part of the ED basin of the N1 atom is marked in light gray.

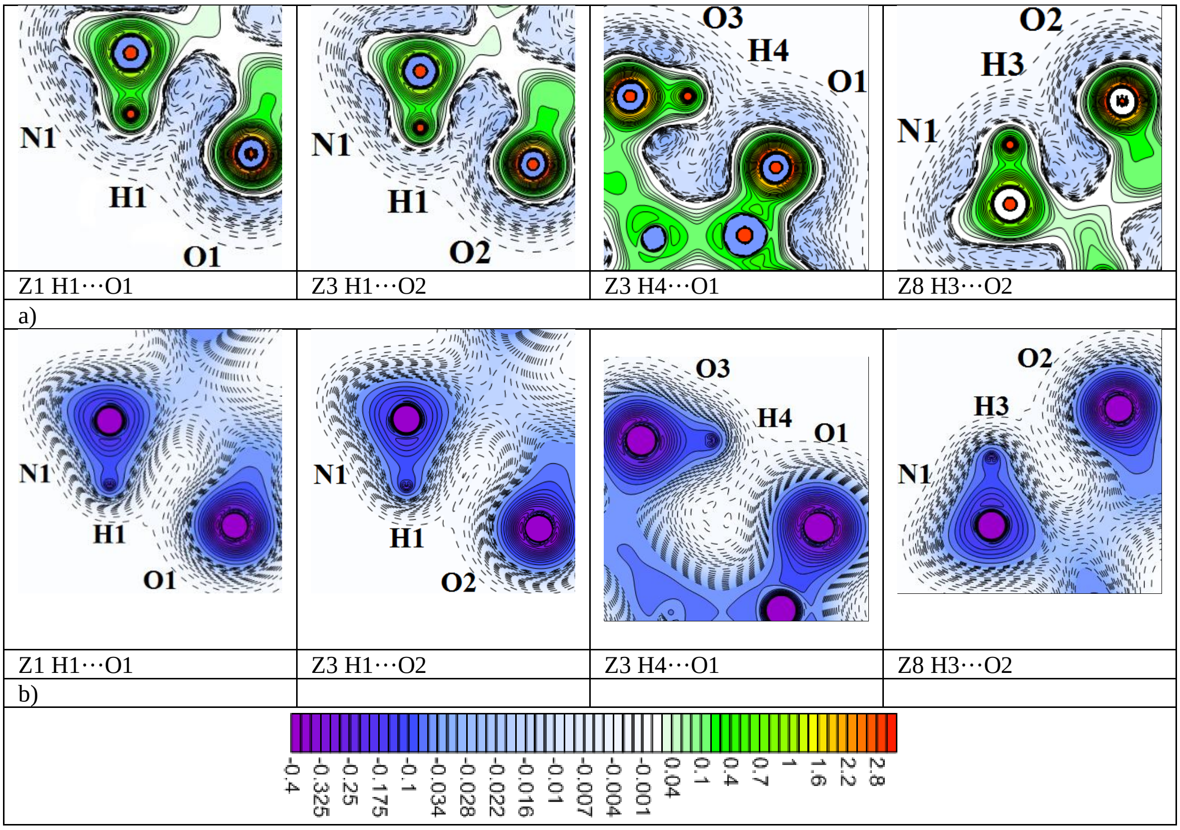

Figure S 11. Distribution of the kinetic (a) and exchange (b) components of internal electronic pressure in the conformers Z1, Z3, and Z8 of serine zwitterion: completed NCIs. Cross-sectional planes pass through the specified atoms. Dashed lines correspond to values from -0.034 to -0.010 a.u. with a step of 0.002 a.u.; from -0.010 to 0 a.u. with a step of 0.001 a.u. Solid lines represent values from -0.400 to -0.050 a.u. with a step of 0.025 a.u.; from 0 to 0.1 a.u. with a step of 0.002 a.u.; from 0.1 to 1.0 a.u. with a step of 0.1 a.u.; and from 1.0 to 3.0 a.u. with a step of 0.2 a.u.

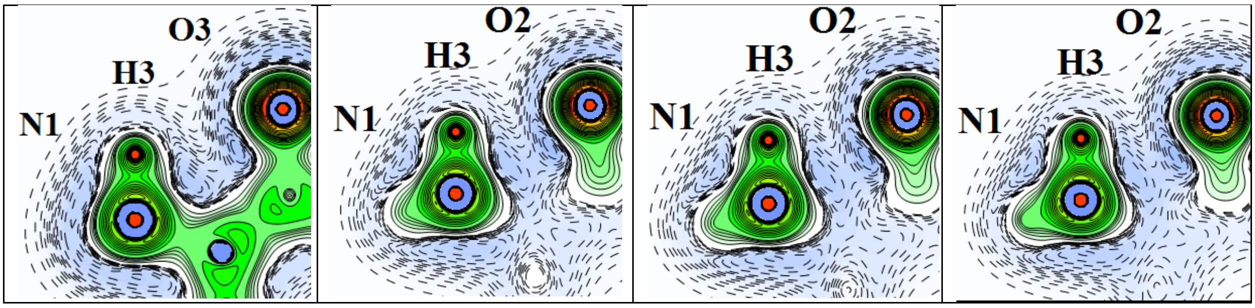

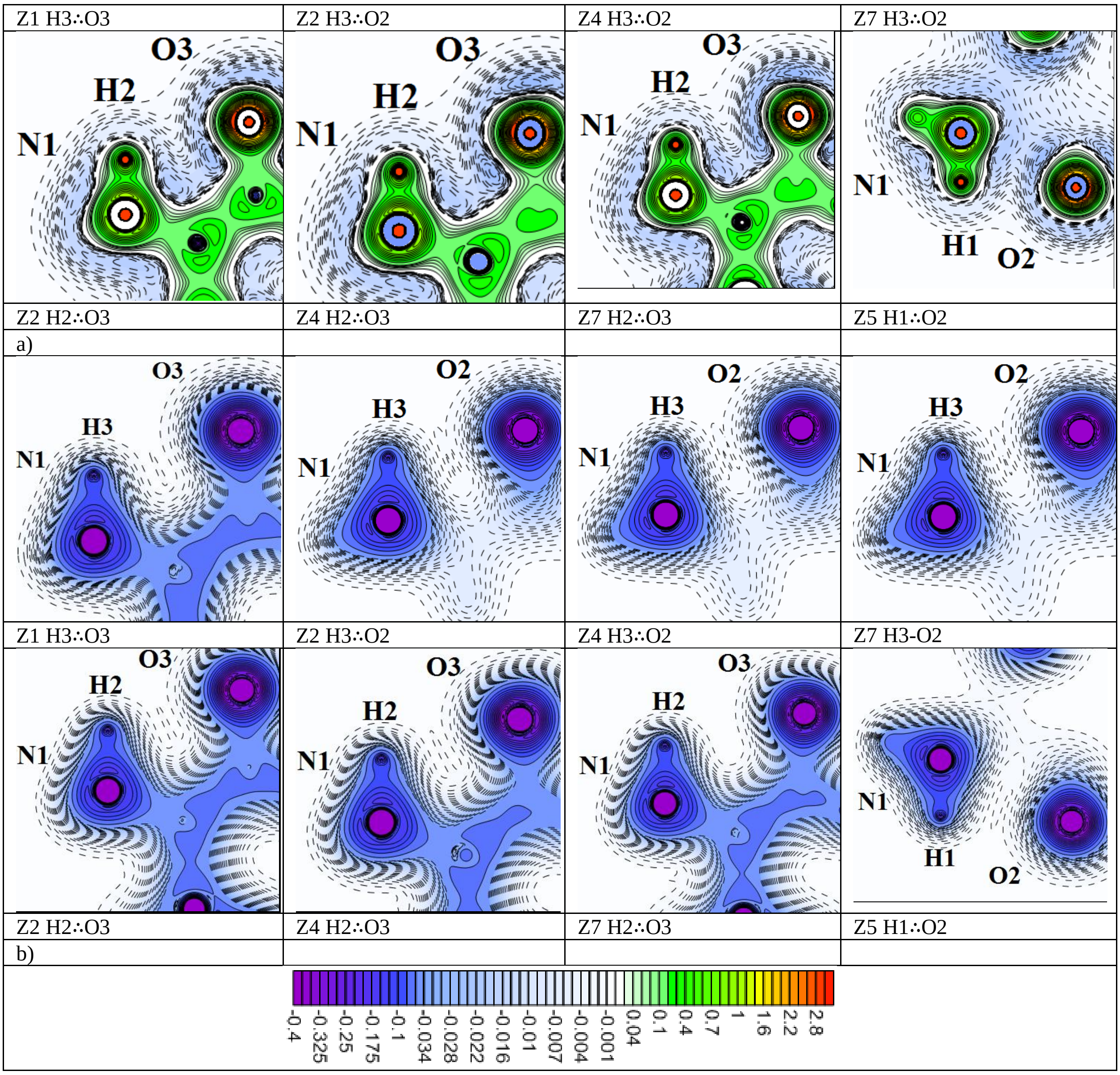

Figure S 12. Distribution of the kinetic (a) and exchange (b) components of internal electronic pressure in the conformers Z1, Z2, Z4, Z5, and Z7 of serine zwitterion: **dynamic** latent NCIs. Cross-sectional planes pass through the specified atoms. Dashed lines correspond to values from -0.034 to -0.010 a.u. with a step of 0.002 a.u.; from -0.010 to 0 a.u. with a step of 0.001 a.u. Solid lines represent values from -0.400 to -0.050 a.u. with a step of 0.025 a.u.; from 0 to 0.1 a.u. with a step of 0.002 a.u.; from 0.1 to 1.0 a.u. with a step of 0.1 a.u.; and from 1.0 to 3.0 a.u. with a step of 0.2 a.u.

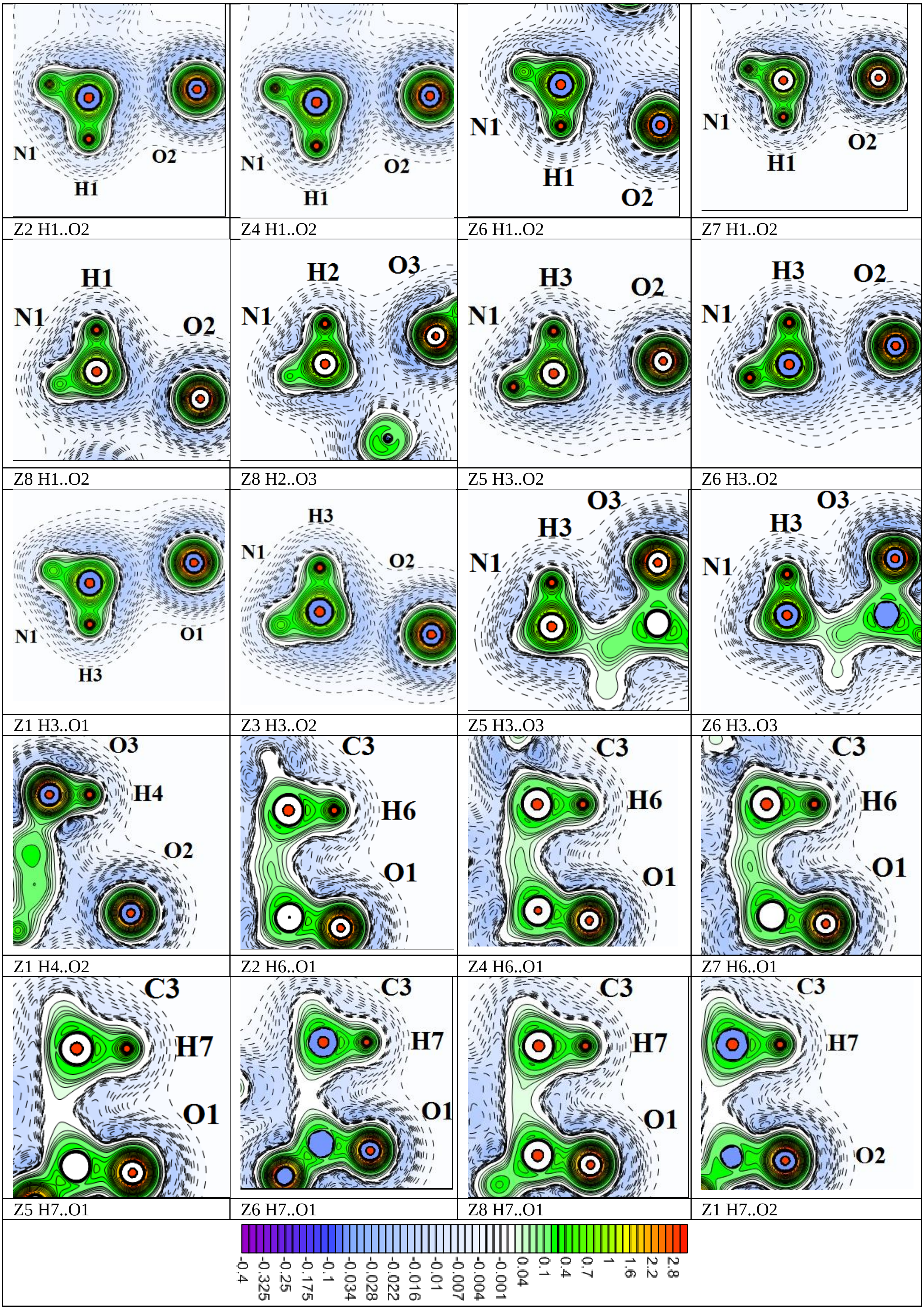

Figure S 13. Distribution of the kinetic component of internal electronic pressure in the conformers Z1-Z8 of the serine zwitterion molecule: **static latent** NCIs. Cross-sectional planes pass through the specified atoms. Dashed lines correspond to values from -0.034 to -0.010 a.u. with a step of 0.002 a.u.; from -0.010 to 0 a.u. with a step of 0.001 a.u. Solid lines represent values from -0.400 to -0.050 a.u. with a step of 0.025 a.u.; from 0 to 0.1 a.u. with a step of 0.002 a.u.; from 0.1 to 1.0 a.u. with a step of 0.1 a.u.; and from 1.0 to 3.0 a.u. with a step of 0.2 a.u.

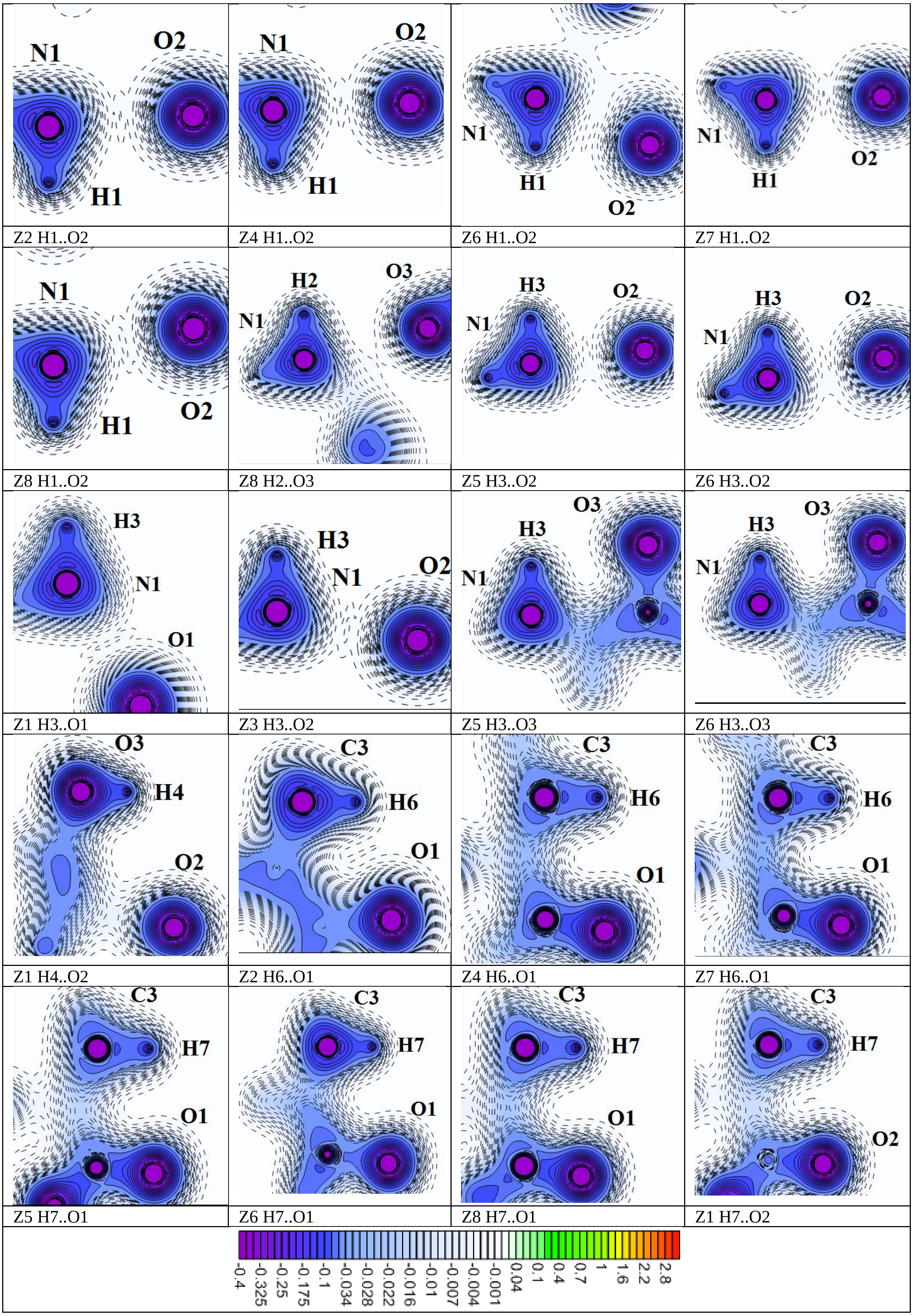

Figure S 14. Distribution of the exchange component of internal electronic pressure in the conformers Z1-Z8 of the serine zwitterion molecule: **static latent** NCIs. Cross-sectional planes pass through the specified atoms. Dashed lines correspond to values from -0.034 to -0.010 a.u. with a step of 0.002 a.u.; from -0.010 to 0 a.u. with a step of 0.001 a.u. Solid lines represent values from -0.400 to -0.050 a.u. with a step of 0.025 a.u.; from 0 to 0.1 a.u. with a step of 0.002 a.u.; from 0.1 to 1.0 a.u. with a step of 0.1 a.u.; and from 1.0 to 3.0 a.u. with a step of 0.2 a.u.

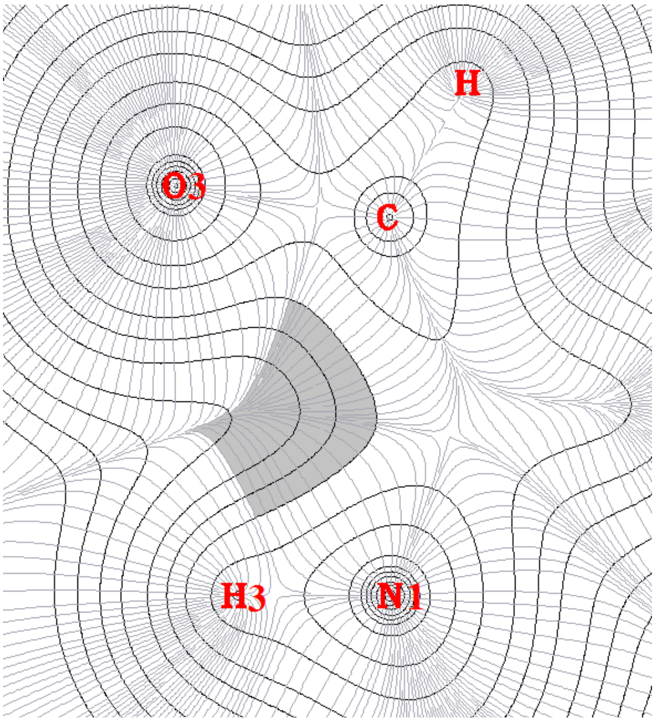

Figure S 15. Distribution of ED and its gradient lines in the plane of atoms N1, H3, and O3 in the nonequilibrium structure of conformer Z6 at a frequency of 187 cm⁻¹, with atomic displacements opposite to the direction of the vectors (-1). Gray color indicates the basins of neighboring atoms: N1 and C3.

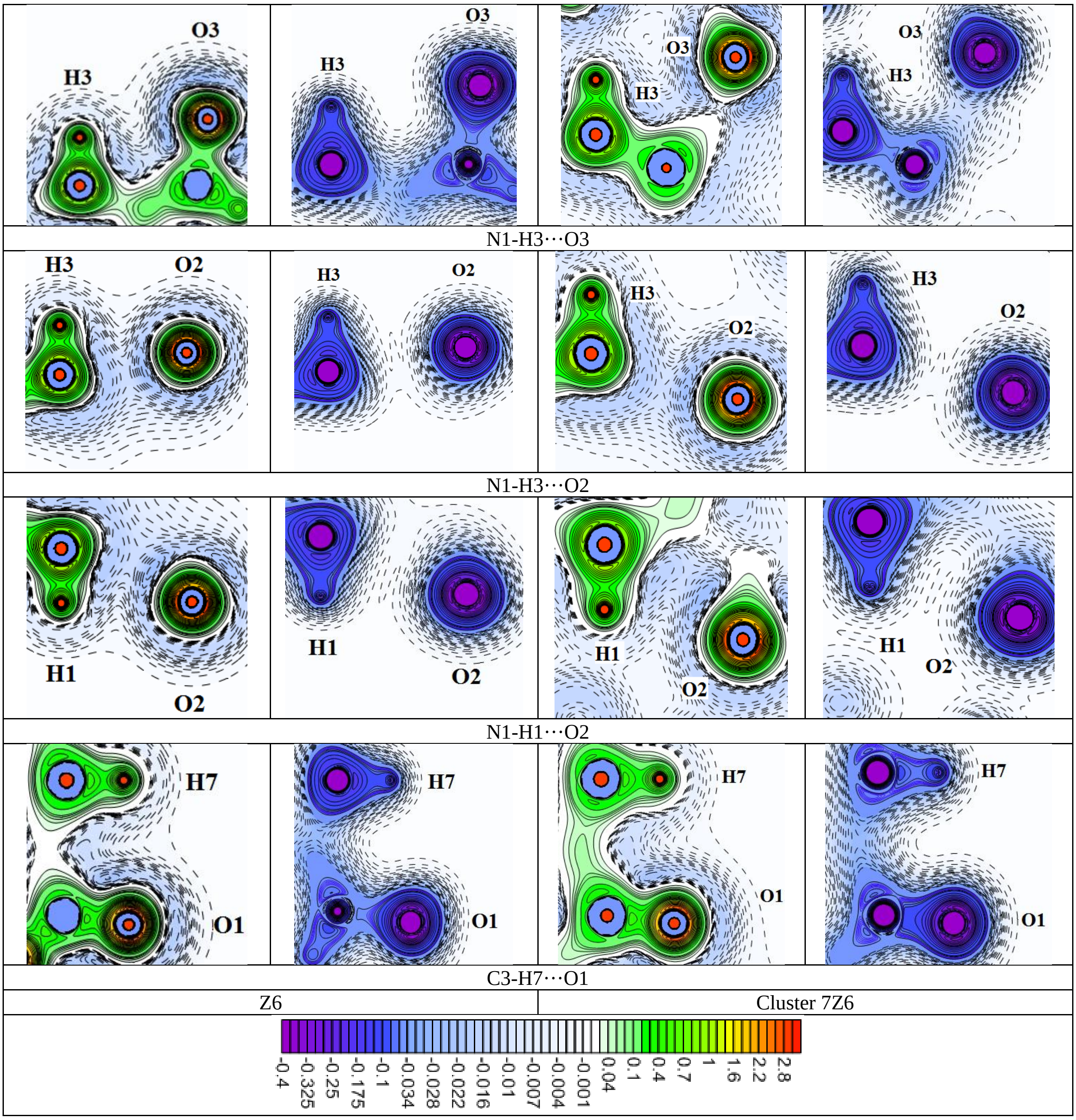

Figure S 16. Intramolecular **incomplete** interactions in the Z6 conformer of serine and the central molecule in the cluster. Electronic continuum pressure and its components: kinetic (left) and exchange (right). Dashed lines correspond to values from -0.034 to -0.010 a.u. with a step of 0.002 a.u.; from -0.010 to 0 a.u. with a step of 0.001 a.u. Solid lines represent values from -0.400 to -0.050 a.u. with a step of 0.025 a.u.; from 0 to 0.1 a.u. with a step of 0.002 a.u.; from 0.1 to 1.0 a.u. with a step of 0.1 a.u.; and from 1.0 to 3.0 a.u. with a step of 0.2 a.u.

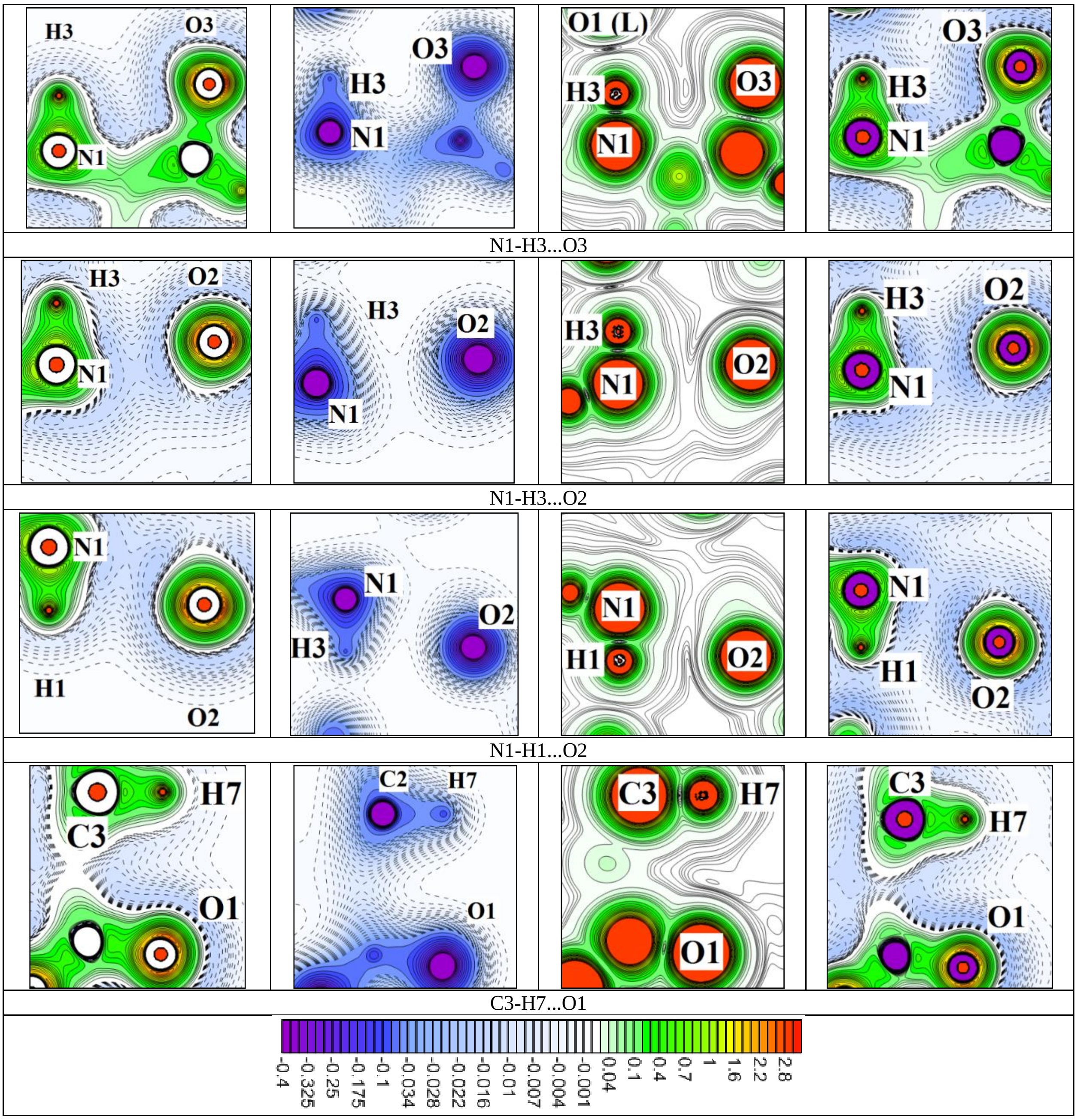

Figure S 17. Intramolecular **incomplete** interactions in the crystal of DL-serine. The electronic continuum pressure and its components are presented in order: kinetic, exchange, electrostatic, and total pressure. Dashed lines correspond to values from -0.034 to -0.010 a.u. with a step of 0.002 a.u.; from -0.010 to 0 a.u. with a step of 0.001 a.u. Solid lines represent values from -0.400 to -0.050 a.u. with a step of 0.025 a.u.; from 0 to 0.1 a.u. with a step of 0.002 a.u.; from 0.1 to 1.0 a.u. with a step of 0.1 a.u.; and from 1.0 to 3.0 a.u. with a step of 0.2 a.u. For the electrostatic component of the pressure, additional isolines are included: from 0.00015 to 0.00055 a.u. with a step of 0.0001 a.u.; from 0.001 to 0.005 a.u. with a step of 0.001 a.u.; and for the H7...O1 interaction, an isoline of 0.00002 a.u.

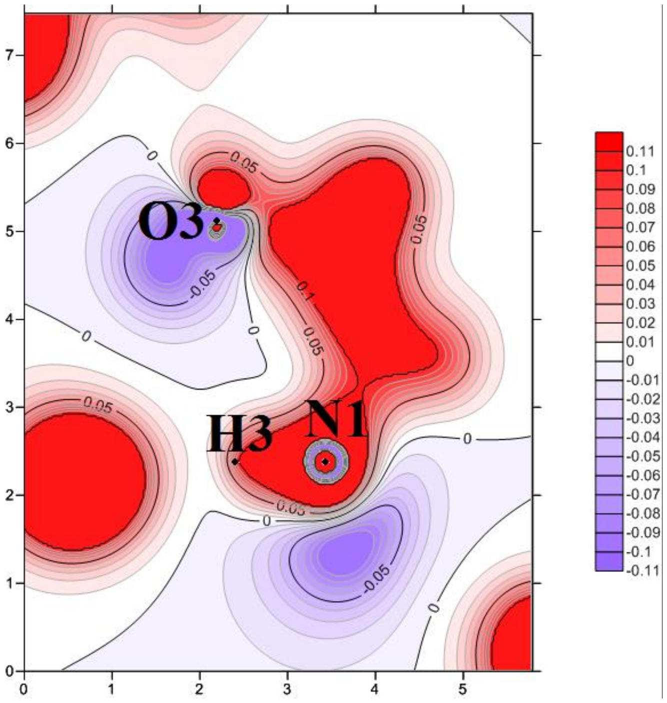

Figure S 18. Interaction N1-H3...O3. Difference in the distribution of the electrostatic potential (EP) between the central molecule in a single-point cluster of 7 molecules and a single-point isolated molecule extracted from the crystal. Atomic nuclei are marked with black dots. Isolines correspond to values from -0.11 to 0.11 a.u. with a step of 0.01 a.u.

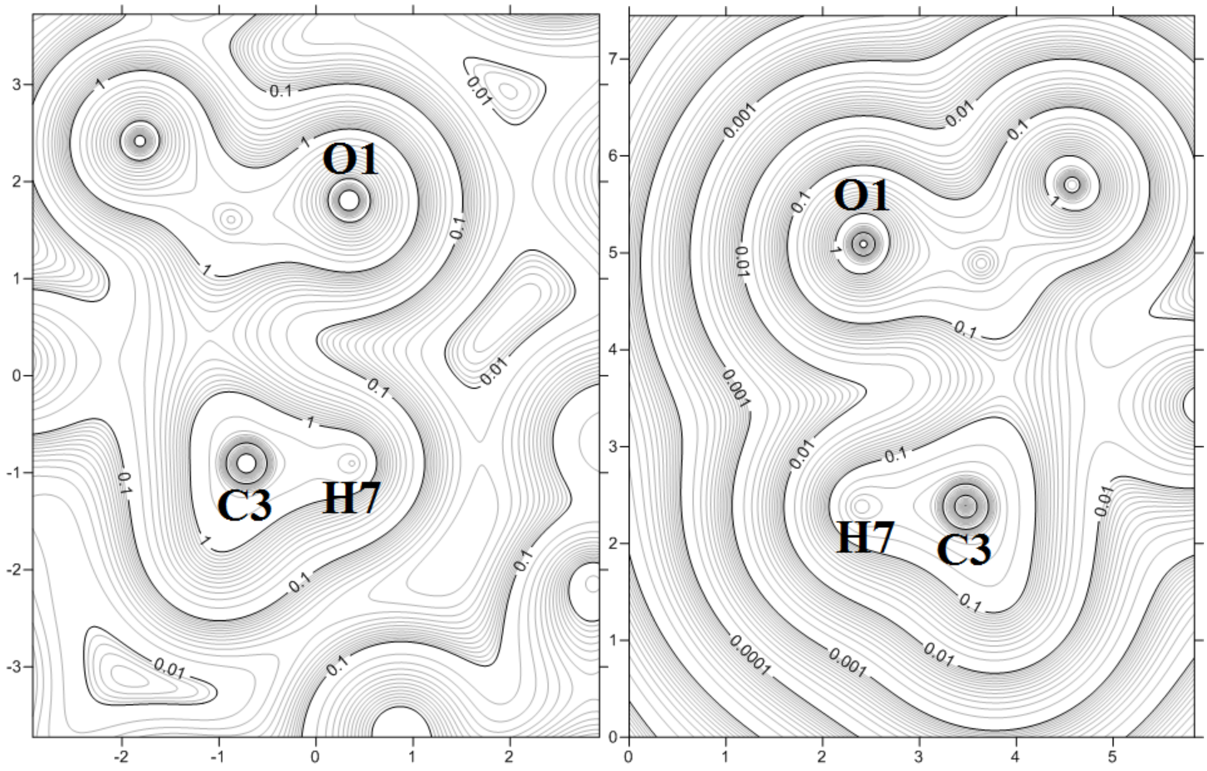

Figure S 19. For the C3-H7...O1 fragment, the experimental ED for the crystal (left) and the theoretical single-point ED for the zwitterion geometry (right), extracted from the crystal, are depicted. Logarithmic isolines with 12 minor intervals are presented.

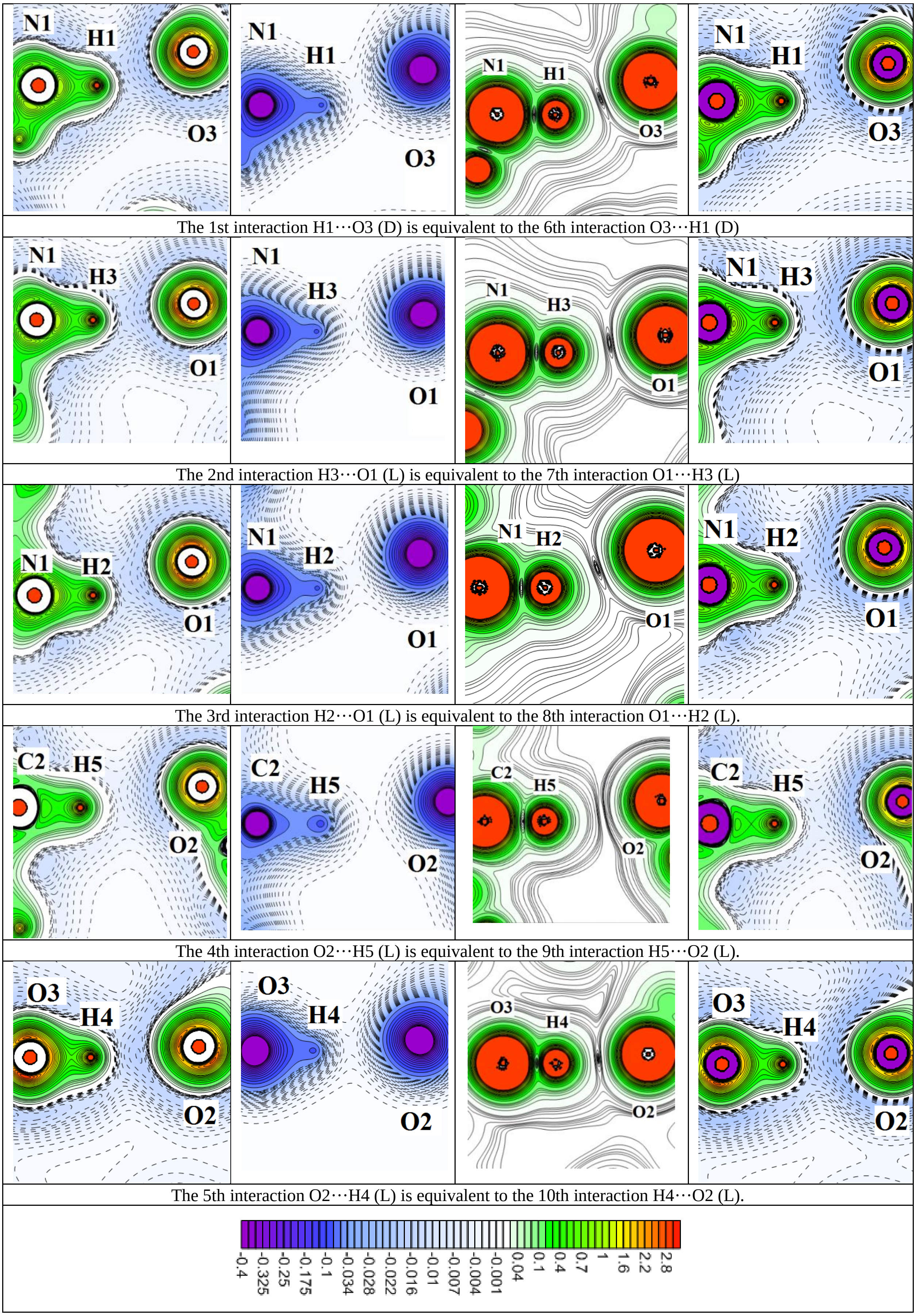

Figure S 20. Intermolecular completed interactions in the DL-serine crystal. The electron continuum pressure and its contributions are presented from left to right: kinetic, exchange, electrostatic, and total pressure. Dashed lines correspond to values ranging from -0.034 to -0.010 a.u. in steps of 0.002 a.u.; from -0.010 to 0 a.u. in steps of 0.001 a.u. Solid lines represent ranges from -0.400 to -0.050 a.u. in steps of 0.025 a.u.; from 0 to 0.1 a.u. in steps of 0.002 a.u.; from 0.1 to 1.0 a.u. in steps of 0.1 a.u.; from 1.0 a.u. to 3.0 a.u. in steps of 0.2 a.u. For the electrostatic part of the pressure, additional isolines are included: from 0.00015 to 0.00055 a.u. in steps of 0.0001 a.u., from 0.001 to 0.005 a.u. in steps of 0.001 a.u.

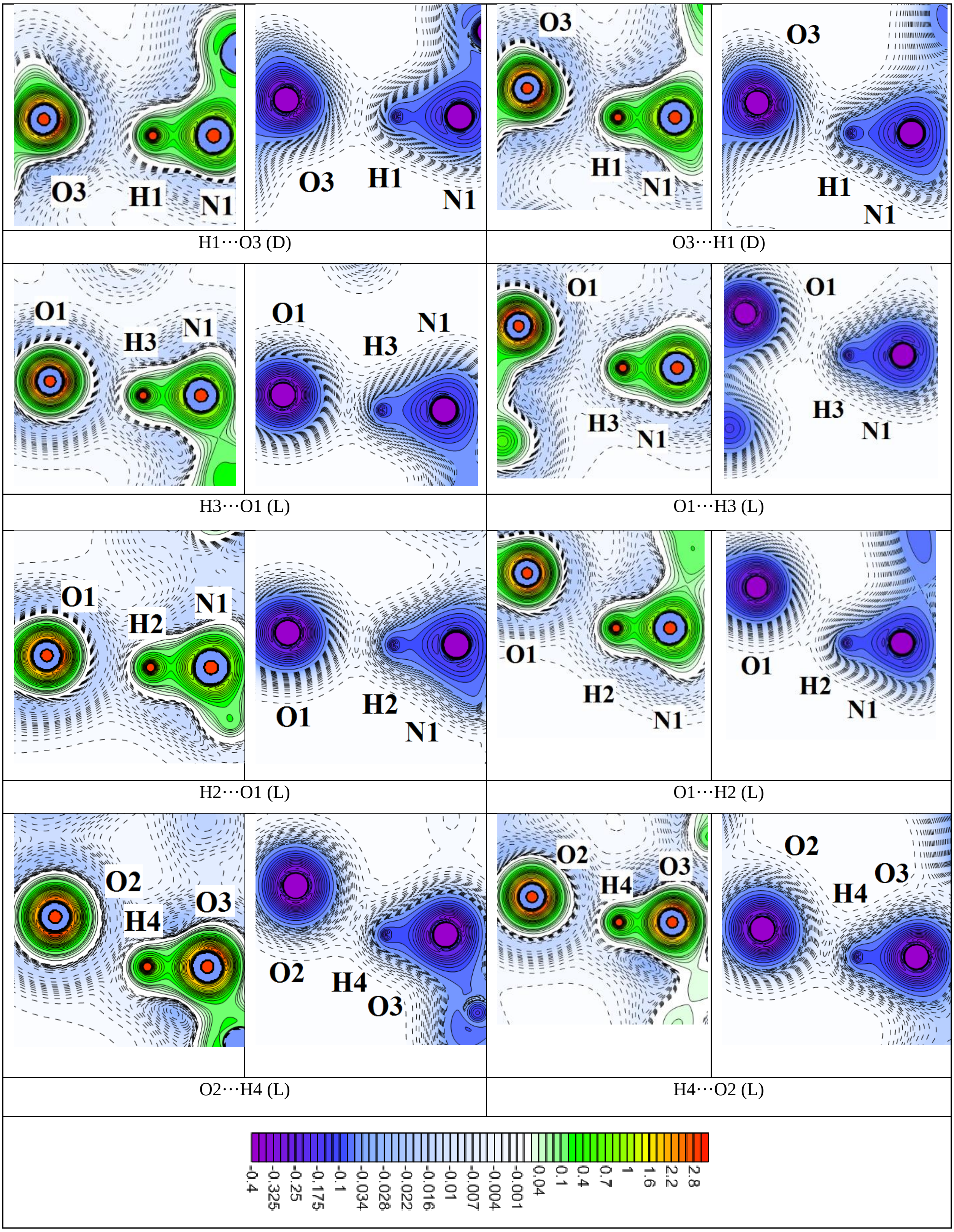

Figure S 21. Intermolecular **completed** interactions in a cluster of 7 serine molecules. The electron continuum pressure and its contributions are presented: left - kinetic, right - exchange. Dashed lines correspond to values ranging from -0.034 to -0.010 a.u. in steps of 0.002 a.u.; from -0.010 to 0 a.u. in steps of 0.001 a.u. Solid lines represent ranges from -0.400 to -0.050 a.u. in steps of 0.025 a.u.; from 0 to 0.1 a.u. in steps of 0.002 a.u.; from 0.1 to 1.0 a.u. in steps of 0.1 a.u.; from 1.0 a.u. to 3.0 a.u. in steps of 0.2 a.u.

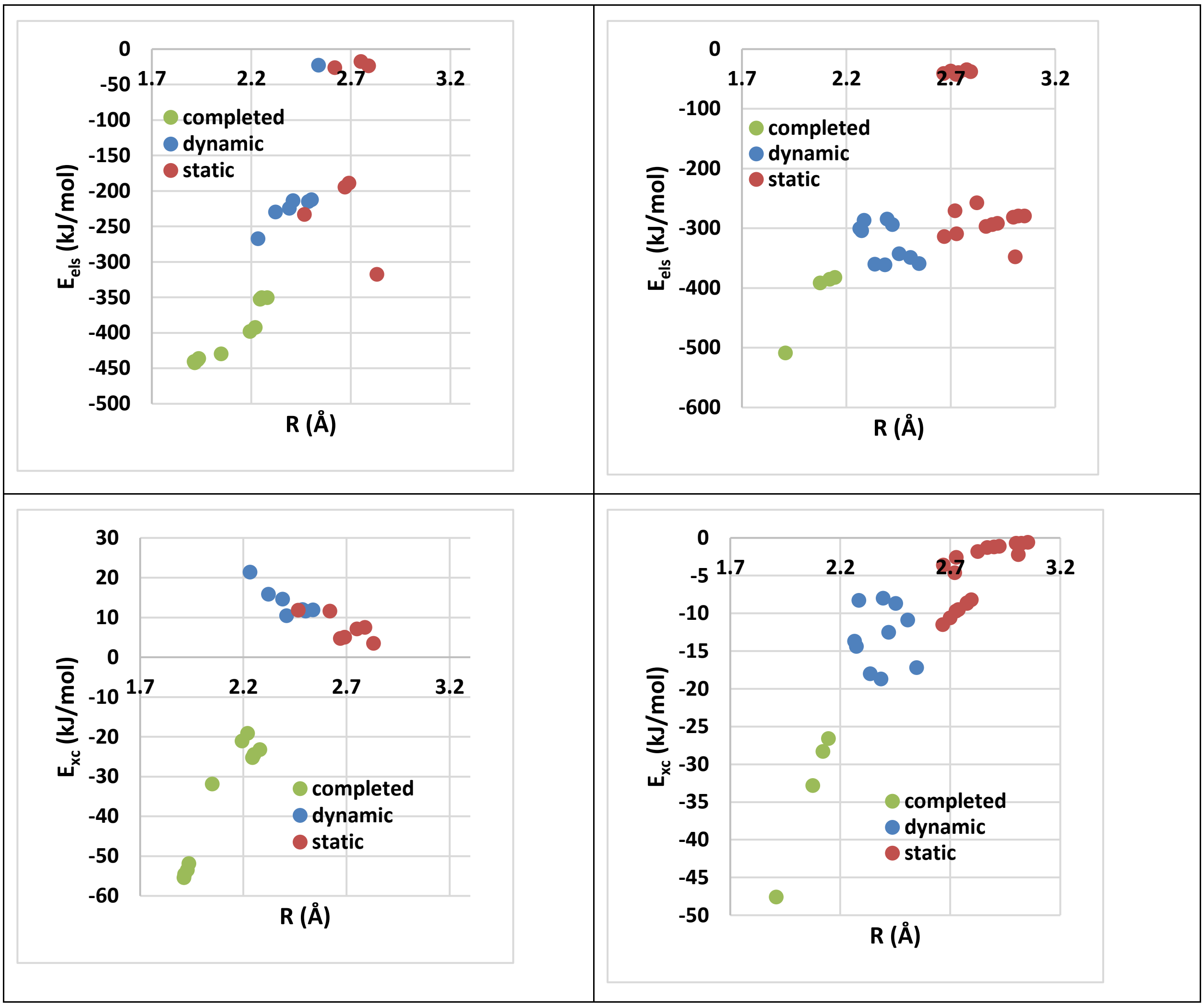

Figure S 22. Electrostatic (E_els, top row) and exchange (E_xc, bottom row) components of interaction energy as a function of interatomic distance R. Left: neutral serine; right: serine zwitterion.

Table S 1. Torsional angles (deg) and relative energies (ΔE, in kJ/mol) of the most stable conformers of the serine molecule based on the results of full geometry optimization (B3LYP/6-311++G**) and frequencies analysis.

Conformer	ΔE	φ [b]	ψ [b]	ω [b]	χ_1 [b]	χ_2 [b]
N1 [a]	0.0	-147.6	18.5	-5.2	56.5	82.1
N2	0.1	35.8	167.3	177.6	-54.5	44.6
N3	1.6	-147.0	17.7	-4.1	-171.5	-54.9
N4	3.7	-19.6	-174.3	179.8	54.4	-46.5
N5	6.3	-93.1	-12.5	1.7	-58.9	178.7
N6	6.5	70.7	70.5	-176.5	59.8	68.2
N7	6.6	-92.5	-14.3	2.9	-57.8	-77.8
N8	6.7	43.2	-26.8	-177.1	-55.4	43.8
N9	9.3	57.8	59.4	-179.0	-176.7	-70.0

a) The electronic energy of the global minimum corresponding to the δL conformation (g-, g+) is -399.0979438 atomic units (a.u.).
b) The torsion angles are defined as follows: φ=(H2-N1-C2-C1), ψ=(N1-C2-C1-O1), ω=(C2-C1-O1-H1), χ_1=(N1-C2-C3-O3), χ_2=(C2-C3-O3-H4).

Table S 2. Topological characteristics of ED, values of interatomic interaction energies E_{int} and their exchangeable V_X^{AB} and electrostatic E_{els}^{AB} components (kJ/mol) for completed NCIs in conformers N of a neutral serine molecule. R(H···Y) - distance between atoms (Å), θ - angle X-H···Y (deg.)

Fragment	N	E_{els}^{AB}	V_x^{AB}	E_{Int}	R	θ	Topological properties of ED		
							ρ_b, a.u.	$\nabla^2\rho_b$, a.u.	$\lvert v_b\rvert/g_b$
O1-H1···N1	N1	-441.9	-54.6	-496.6	1.916	124.9	0.036	0.105	1.056
	N3	-436.2	-51.9	-488.1	1.936	110.2	0.034	0.104	1.037
	N5	-440.2	-55.4	-495.7	1.913	106.6	0.036	0.105	1.055
	N7	-438.3	-53.5	-491.8	1.929	91.5	0.035	0.104	1.043
O3-H4···N1	N2	-352.3	-25.2	-377.5	2.245	112.9	0.019	0.072	0.872
	N4	-350.3	-23.2	-373.5	2.280	108.2	0.018	0.071	0.867
	N8	-350.5	-24.5	-375.0	2.252	96.7	0.019	0.071	0.875
O3-H4···O2	N3	-429.5	-31.9	-461.4	2.049	97.1	0.022	0.077	0.923
	N6	-397.9	-21.1	-419.0	2.194	125.0	0.017	0.059	0.905
	N9	-392.5	-19.1	-411.7	2.220	108.6	0.016	0.057	0.899

Table S 3. The calculated values of atomic energies E_{Int}^H and E_{Int}^Y, interatomic interaction energies E_{int} and their exchange V_X^{AB} and electrostatic E_{els}^{AB} components (kJ/mol) for **latent** NCIs in conformers N of a neutral serine molecule. R(H...Y) - distance between atoms (Å), θ - angle X-H...Y (deg.)

Fragment	N	E_{els}^{AB}	V_x^{AB}	E	R	Θ	E_{Int}^H	E_{Int}^Y	ΔE_{Int}^{H*} N_i - N_j	ΔE_{Int}^{Y*} N_i - N_j	ν cm^{-1**}
N1-H3∴O3	N1	-214.8	12.0	-226	2.487	125.4	-1322.2	-197104.7	+1.6 (N1- N3)	-17.7 (N1- N3)	58+<br>102-<br>218+
O3-H4..O2	N1	-317.4	3.5	-321	2.831	102.5	-1225.1	-196982.9	-4.6 (N1- N7)	-10.5 (N1- N7)	58-
C3-H7..O2	N1	-26.1	11.6	-37	2.619	93.5	-1463.5	-196983.0	+3.2 (N1- N7)	-10.5 (N1- N7)	292-
N1-H2..O2	N2	-233.0	11.8	-244	2.467	114.0	-1332.7	-197004.6	-3.9 (N2- N7)	-32.2 (N2- N7)	238-
C3-H6..O1	N2	-17.5	7.1	-24	2.751	114.0	-1469.5	-197154.3	+0.4 (N2- N7)	-43.1 (N2- N7)	187-
N1-H2∴O2	N4	-267.2	21.4	-288	2.233	100.5	-1327.0	-197001.2	+1.8 (N4- N7)	-28.8 (N4- N7)	223-<br>324+<br>279+<br>366-<br>481-
C3-H7∴O1	N4	-22.4	11.9	-34	2.537	124.6	-1467.6	-197152.1	-0.9 (N4- N7)	-40.7 (N4- N7)	233+<br>279-<br>366-
N1-H3∴O3	N5	-229.4	15.8	-245	2.322	108.0	-1319.7	-197113.9	+4.1 (N5- N3)	-26.9 (N5- N3)	125+<br>195-<br>317+<br>325-
C3-H6..O2	N5	-23.5	7.5	-31	2.789	100.2	-1463.5	-196969.2	+1.4 (N5- N7)	+3.1 (N5- N7)	-
N1-H3..O1	N6	-188.9	5.0	-193	2.691	124.1	-1341.3	-197154.8	-17.5 (N6- N3)	-29.2 (N6- N3)	-
N1-H2∴O3	N6	-212.3	11.6	-223	2.502	126.5	-1337.4	-197082.0	+3.8 (N6- N9)	-0.3 (N6- N9)	152-<br>222-<br>254-
N1-H3∴O3	N7	-224.3	14.6	-238	2.391	92.6	-1319.3	-197111.7	+4.5 (N7- N3)	-24.7 (N7- N3)	122+<br>191-<br>314-
N1-H2∴O1	N8	-213.4	10.4	-223	2.410	92.9	-1332.0	-197153.7	-3.2 (N8- N7)	-42.3 (N8- N7)	280-<br>281-<br>329-
N1-H3..O1	N9	-194.6	4.7	-199	2.670	134.5	-1335.4	-197154.9	+1.5 (N9- N2)	-0.6 (N9- N2)	284-

*) $\Delta E_{Int}^{H,Y}$ - difference of atomic energies of H (in conformers N_i and N_j) and Y (in conformers N_i and N_j).
**) ν, cm^{-1} - frequencies of vibrations at which bonding paths are formed in nonequilibrium structures; + and - indicate displacements of atoms along and against the direction of vectors, respectively.

Table S 4. Topological characteristics of the electron density, interatomic interaction energy values (E_{int}) and their exchange (V_X^{AB}) and electrostatic (E_{els}^{AB}) components (kJ/mol) for completed NCIs in the Z conformers of the serine zwitterion molecule. R(H⋯Y) - distance between atoms (Å), θ(X-H⋯Y) angle (degrees).

Fragment	Z	E_{els}^{AB}	V_X^{AB}	E_{Int}	R	θ	Topological characteristics		
							ρ_b. a.u.	$\nabla 2\rho_b$. a.u.	|vb|/gb
N1-H1⋯O1 a)	Z1	-385.3	-28.3	-413.5	2.121	108.3	0.022	-0.023	0.834
N1-H1⋯O2 a)	Z3	-382.3	-26.6	-408.9	2.146	107.9	0.021	-0.022	0.827
N1-H3⋯O2 a)	Z8	-391.1	-32.8	-423.9	2.075	111.2	0.024	-0.024	0.851
O3-H4⋯O1 a)	Z3	-508.7	-47.6	-556.3	1.909	109.1	0.030	-0.025	0.988

Table S 5. Values of atomic energies E_{Int}^H and E_{Int}^Y, interatomic interaction energies E_{int} and their exchangeable V_X^{AB} and electrostatic E_{els}^{AB} components (kJ/mol) for **latent** NCIs in the zwitterion conformers of serine molecule. R(H⋯Y) - distance between atoms (Å), θ - angle X-H⋯Y (deg.)

Fragment	Z	E_{els}^{AB}	V_x^{AB}	E_{Int}	R	Θ	E_{Int}^H	E_{Int}^Y	ΔE_{Int}^H Z_i - Z_j	ΔE_{Int}^Y Z_i - Z_j	ν, cm^{-1**}
O3-H4..O2	Z1	-347.6	-2.2	-349.8	3.009	109.1	-1208.4	-196886.6	-34.9 (Z1-Z4)	-20.1 (Z1-Z4)	59.73 -
N1-H3∴O3	Z1	-293.7	-12.5	-306.3	2.420	107.0	-1210.1	-197146.3	-31.1 (Z1-Z3)	-76.6 (Z1-Z3)	59.73 + 81.28 + 195.15 - 279.23 +
N1-H3..O1	Z1	-281.2	-0.7	-281.8	3.000	59.4	-1210.1	-196923.5	-31.1 (Z1-Z3)	-57.0 (Z1-Z4)	-
C3-H7..O2	Z1	-40.6	-11.5	-52.1	2.666	92.5	-1455.3	-196886.6	7.0 (Z1-Z1)	-20.1 (Z1-Z4)	279.23 -
N1-H3∴O2	Z2	-360.8	-18.7	-379.5	2.265	101.2	-1213.0	-196917.6	-34.0 (Z2-Z3)	-74.6 (Z2-Z4)	193.93 + 273.98 - 331.61 -
N1-H2∴O3	Z2	-300.1	-13.7	-313.8	2.385	106.8	-1196.9	-197186.0	-17.9 (Z2-Z3)	-116.3 (Z2-Z3)	109.54 - 193.93 + 273.98 + 331.61 -
N1-H1..O2	Z2	-291.5	-1.1	-292.5	2.923	65.2	-1198.9	-196917.6	13.1 (Z2-Z1)	-51.1 (Z2-Z4)	-
C3-H6..O1	Z2	-37.6	-8.2	-45.8	2.796	93.1	-1455.7	-196874.4	4.5 (Z2-Z1)	13.9 (Z2-Z4)	-
N1-H3..O2	Z3	-279.3	-0.7	-280.0	3.024	59.1	-1189.7	-196941.1	-10.7 (Z3-Z3)	-51.1 (Z3-Z4)	-
N1-H3∴O2	Z4	-360	-18.0	-378.0	2.273	100.4	-1213.7	-196915.9	-34.7 (Z4-Z3)	-49.4 (Z4-Z4)	207.29 + 274.00 - 342.58 -
N1-H2∴O3	Z4	-304	-14.4	-318.5	2.336	106.2	-1200.0	-197186.7	-21.0 (Z4-Z3)	-117.0 (Z4-Z3)	127.03 + 198.59 + 274.00 + 342.58 -
N1-H1..O2	Z4	-293.4	-1.2	-294.6	2.899	66.2	-1203.8	-196915.9	8.2 (Z4-Z1)	-49.4 (Z4-Z4)	-
C3-H6..O1	Z4	-34.5	-8.7	-43.2	2.776	93.2	-1456.1	-196866.5	4.1 (Z4-Z1)	(0.0) (Z4-Z4)	-
N1-H3∴O3	Z5	-284.5	-8.0	-292.5	2.507	100.5	-1214.8	-197140.7	-35.8 (Z5-Z3)	-71.0 (Z5-Z3)	102.76 + 196.29 -
N1-H3..O2	Z5	-309.3	-2.6	-311.9	2.728	74.6	-1214.8	-196888.3	-35.8 (Z5-Z3)	-21.8 (Z5-Z4)	-
N1-H1∴O2	Z5	-348.7	-10.9	-359.6	2.395	92.6	-1210.2	-196888.3	1.8 (Z5-Z1)	-21.8 (Z5-Z4)	38.67 - 144.82 + 196.29 + 262.62 -
C3-H7..O1	Z5	-36.6	-10.6	-47.2	2.700	92.6	-1456.2	-196846.3	-0.9 (Z5-Z1)	20.2 (Z5-Z4)	336.12 -
N1-H3..O3	Z6	-270.3	-4.6	-274.9	2.721	98.1	-1198.9	-197135.3	-19.9 (Z6-Z3)	-65.6 (Z6-Z3)	394.27 -
N1-H3..O2	Z6	-313.8	-3.6	-317.3	2.669	77.7	-1198.9	-196896.9	-19.9 (Z6-Z3)	-30.4 (Z6-Z4)	-
N1-H1∴O2	Z6	-342.5	-8.7	-351.1	2.453	89.4	-1200.6	-196896.9	11.4 (Z6-Z1)	-30.4 (Z6-Z4)	46.21 - 105.05 +
C3-H7..O1	Z6	-42.1	-9.7	-51.8	2.726	91.9	-1455.0	-196844.7	0.3 (Z6-Z1)	21.8 (Z6-Z4)	329.73 -
N1-H3∴O2	Z7	-359	-17.2	-376.2	2.285	99.5	-1204.8	-196920.8	-25.8 (Z7-Z3)	-54.3 (Z7-Z4)	151.43 + 175.79- 232.40 -

N1-H2∴O3	Z7	-286.3	-8.3	-294.6	2.548	103.1	-1186.7	-197181.4	-7.7 (Z7-Z3)	-111.7 (Z7-Z3)	58.10 + 73.87 - 175.79 + 232.40 +
N1-H1..O2	Z7	-296.8	-1.3	-298.1	2.869	67.6	-1196.7	-196920.8	15.3 (Z7-Z1)	-54.3 (Z7-Z4)	-
C3-H6..O1	Z7	-39.4	-9.5	-48.9	2.737	95.4	-1455.1	-196865.9	5.1 (Z7-Z1)	0.6 (Z7-Z4)	-
N1-H2..O3	Z8	-257	-1.8	-258.8	2.825	83.6	-1197.1	-197134.2	-18.1 (Z8-Z3)	-64.5 (Z8-Z3)	-
N1-H1..O2	Z8	-279.1	-0.6	-279.7	3.053	56.4	-1198.3	-196891.0	13.7 (Z8-Z1)	-24.5 (Z8-Z4)	-
C3-H7..O1	Z8	-34.6	-8.6	-43.2	2.776	92.4	-1456.9	-196852.6	-1.6 (Z8-Z1)	13.9 (Z8-Z4)	-

*) The E_{Int}^{H} and E_{Int}^{Y} values for the conformers compared were E_{Int}^{H4}(Z4) = -1173.5 kJ/mol, E_{Int}^{H2}(Z3) = -1179. 0 kJ/mol, E_{Int}^{H6}(Z3) = -1466.0 kJ/mol, E_{Int}^{H7}(Z3) = -1462.3 kJ/mol, E_{Int}^{O1}(Z4) = -196866.5 kJ/mol, E_{Int}^{O1}(Z3) = -197069.7 kJ/mol.

(*) $\Delta E_{Int}^{H,Y}$ is the difference of atomic energies of H (in conformers Z_i and Z_j) and Y (in conformers Z_i and Z_j). The values of $\Delta E_{Int}^{H,Y}$ in conformers N_j are given in Table S 3.

(**)ν, cm^{-1} are the frequencies of vibrations at which bonding paths are formed in nonequilibrium structures; + and - indicate the displacements of atoms along and against the direction of vectors, respectively.

Table S 6. Topological characteristics of ED for **completed** intermolecular NCIs in the crystal of DL-serine. R(H...Y) - distance between atoms (Å), θ - X-H...Y angle (deg.).

Fragment	R	Θ	Topological characteristics		
			ρ_b, a.u.	$\nabla 2\rho_b$, a.u.	|vb|/gb
H1···O3 (D)<br>O3···H1 (D)	1.767	160.5	0.031	0.143	0.897
H3···O1 (L)<br>O1···H3 (L)	1.820	170.6	0.013	0.055	0.924
H2···O1 (L)<br>O1···H2 (L)	1.807	161.2	0.014	0.059	0.925
O2···H5 (L)<br>H5···O2 (L)	2.315	170.7	0.016	0.058	0.836
O2···H4 (L)<br>H4···O2 (L)	1.762	174.6	0.019	0.077	0.968

Table S 7. Topological characteristics of the ED for completed intermolecular NCIs in the 7-molecule serine cluster. R(H...Y) represents the distance between atoms (Å), and θ is the X-H...Y angle (degrees).

Fragment	R	Θ	Topological characteristics		
			ρ_b, a.u.	$\nabla 2\rho_b$, a.u.	|vb|/gb
H1···O3 (D)	1.865	171.4	0.031	-0.026	0.969
O3···H1 (D)	1.725	162.2	0.043	-0.034	1.078
H3···O1 (L)	1.707	171.2	0.046	-0.032	1.132
O1···H3 (L)	2.101	158.1	0.019	-0.016	0.886
H2···O1 (L)	1.774	173.5	0.036	-0.032	0.992
O1···H2 (L)	1.971	148.2	0.023	-0.023	0.855
O2···H4 (L)	1.707	151.6	0.044	-0.035	1.077
H4···O2 (L)	1.683	163.7	0.047	-0.034	1.122
